# Characterization of the Signaling Pathways Activated by KCl-Induced RTK Stimulation in Guinea Pig Airways

**DOI:** 10.3390/biology14111557

**Published:** 2025-11-06

**Authors:** Eva Herrera-Alcibar, Edgar Flores-Soto, Ruth M. López, Enrique F. Castillo, Patricia Campos-Bedolla, Verónica Carbajal, Bettina Sommer

**Affiliations:** 1 Departamento de Investigación en Hiperreactividad Bronquial, Instituto Nacional de Enfermedades Respiratorias “Ismael Cosío Villegas”, Mexico City 14080, Mexico; ema.he.alci@gmail.com; 2 Sección de Estudios de Posgrado e Investigación, Escuela Superior de Medicina, Instituto Politécnico Nacional, Mexico City 11340, Mexico; rmelopezm@ipn.mx (R.M.L.); ecastilloh@ipn.mx (E.F.C.); 3 Departamento de Farmacología, Facultad de Medicina, Universidad Nacional Autónoma de Mexico, Mexico City 04360, Mexico; edgarfloressoto@yahoo.com.mx; 4 Unidad Médica en Investigación en Enfermedades Neurológicas, Hospital de Especialidades “Bernardo Sepúlveda”, Instituto Mexicano del Seguro Social (IMSS), Mexico City 06720, Mexico; camposbedollap@hotmail.com

**Keywords:** KCl, RTK, MAPK, ERK, ROCK

## Abstract

**Simple Summary:**

Airway smooth muscle (ASM) adjusts airway diameter in response to an ample variety of stimuli; however, the physical causes remain poorly understood. To fully comprehend complex pathological states like asthmatic ASM and its relation to airway hyperresponsiveness, basic ASM physiology needs to be thoroughly understood. Agonists (histamine, acetylcholine, hydroxitriptamine, etc.) activate well known cell signaling, while KCl is used because of its capacity to depolarize the ASM membrane and induce contraction by bypassing agonist-induced second messenger signaling cascades. In vitro, KCl-induced ASM depolarization activates receptor tyrosine kinases (RTKs). It is worth mentioning that this approach emulates airway smooth muscle signaling pathways activated by physical circumstances (mucus osmolarity, tidal volume, PM2.5). It is known that RTKs are single subunit receptors, and ligands binding to their extracellular domain induce receptor dimerization, initiating downstream signal transduction pathways such as the MAP kinase signaling cascade. In this sense, KCl-induced RTK stimulation is a useful strategy to dissect the signaling pathways involved in ASM contraction. The study of these pathways might contribute to uncovering a potential therapeutic approach for chronic respiratory diseases like asthma. Therefore, we theorized that, in ASM, a low concentration (20 mM) of KCl activates RTKs and their downstream signaling cascades (i.e., MAPK-ERK and ROCK) and studied whether these cascades participate in KCl-induced ASM contraction.

**Abstract:**

We found that, in guinea pig airway smooth muscle, the pharmacological inhibition of RTKs significantly decreased the contraction induced by 20 mM KCl. We observed that MEK pharmacological inhibitors diminished the contraction induced by 20 mM KCl, but not that induced by 60 mM. On the other hand, ERK inhibitors also altered the contraction generated by 20 mM KCl. When a ROCK inhibitor was tested, we found that it significantly inhibited the KCl-induced contraction. These results were complemented with Western blot experiments, and a decrease in ERK phosphorylation was noticed when the RTKs were inhibited. When MEK and ERK inhibitors were used, we also observed a decrease in ERK phosphorylation. In the case of MYPT1, its phosphorylation decreased when RTK, MEK, and ROCK inhibitors were used. In conclusion, we found that, in guinea pig airway smooth muscle, the contraction induced by 20 mM KCl includes the activation of RTKs and, in turn, MEK-ERK and ROCK.

## 1. Introduction

Airway smooth muscle (ASM) adjusts airway diameter in response to an ample variety of stimuli, including neurotransmitters, inflammatory mediators, inspired air volume and temperature, stress and strain, airborne particles, and other factors [1,2]. To fully comprehend a complex pathological state like ASM hyperresponsiveness, basic ASM physiology needs to be thoroughly understood. Regarding ASM contraction physiology, terms like pharmacomechanical and electromechanical coupling are commonly used. Although not strictly independent in a real-time contraction event, they have been conceptualized here as pharmacomechanical, that is, a membrane-potential-independent, but second-messenger-dependent contraction; and electromechanical, that is, a membrane-potential-dependent, but second-messenger-independent contraction [3]. Because these couplings are considered independent, certain pharmacological tools have been employed in vitro to study each contraction mode. Agonists activating their cognate Gαq-coupled receptor (e.g., histamine to activate H1 receptors, carbachol to activate M3 receptors) have been employed to study pharmacomechanical coupling, while KCl has been used to investigate electromechanical coupling, allegedly because of its capacity to depolarize the ASM membrane and induce contraction by bypassing Gαq-activated second-messenger signaling cascades. Continuous and systematic research over many years revealed that KCl can activate the RhoA/Rho kinase (ROCK) pathway [4,5], inducing the calcium sensitization phenomenon. In most smooth muscles, Ca^2+^ sensitization is carried out by the monomeric G protein RhoA and its downstream effector, ROCK. The latter phosphorylates myosin light chain phosphatase (MLCP) on its targeting subunit (MYPT1), inhibiting its activity and promoting sustained contraction without noticeable changes in intracellular Ca^2+^ concentrations [3]. In addition, it has been demonstrated that by modifying ASM membrane potential, both 20 mM and 60 mM KCl contribute to enhancing contraction responses to cholinergic stimulation [6]. Seemingly, ASM membrane depolarization through high K^+^ addition contributes to open L-type voltage-dependent Ca^2+^ channels (L-VDCC), induces calcium sensitization, and facilitates contractile responses to cholinergic agonists. These findings suggest that not only are both coupling modes interdependent, but a slightly depolarized ASM membrane might predispose the airway to hyperresponsiveness—a cardinal asthma symptom.

In vitro KCl-induced ASM depolarization remains a useful pharmacological approach to study ASM contraction. It has been found that depolarization triggers mitogen and protein-activated kinases that, in turn, phosphorylate other transducer proteins like Transient Receptor Potential Vanilloid 1 (TRPV1) [7]. TRPV1 is expressed in ASM [8], and recent studies point out that its blockade significantly diminishes airway hyperresponsiveness, airway inflammation, and ASM remodeling in a chronic murine asthma model [9]. These findings imply that KCl-induced depolarization is not a simple phenomenon and might be considered a complex signaling cascade that deserves abundant and rigorous investigation. In this regard, research carried out in rat caudal arterial smooth muscle indicates that this depolarization triggers a genistein-sensitive tyrosine kinase involved in ROCK activation [10]; furthermore, genistein has been used as an unspecific inhibitor for receptor tyrosine kinases (RTKs) [11,12]. Most RTKs are single subunit receptors and ligands binding to their extracellular domain to induce receptor dimerization, initiating downstream signal transduction pathways, such as the MAP (mitogen-activated protein) kinase signaling cascade [13] that, according to the available literature, are activated by high potassium solutions [14,15]. Their activation triggers signaling cascades that regulate key cellular processes such as proliferation, differentiation, migration, and survival. Among the downstream signaling pathways of RTKs, the mitogen-activated protein kinase/extracellular signal-regulated kinase (MAPK/ERK) [13,16] and RhoA/ROCK [17] pathways stand out. MEK (mitogen-activated kinase kinase) is a central component of the MAP kinase pathway, responsible for phosphorylating and activating ERK1/2. This cascade is involved in ASM hyperplasia, hypertrophy, and remodeling [18], phenomena frequently observed in asthma. On the other hand, ROCK, activated by RhoA, directly regulates ASM contraction through the inhibition of MLCP, promoting the maintenance of contraction through calcium sensitization mechanisms [3]. Furthermore, ROCK is involved in cytoskeletal reorganization, cell migration, and the expression of genes related to the contractile phenotype [19]. The integration of signals from RTKs and the activation of pathways such as MEK/ERK and RhoA/ROCK represent a key point in understanding the molecular mechanisms of ASM contraction, inflammation, and tissue remodeling. In this sense, KCl-induced RTK stimulation is an accessible strategy to dissect signaling pathways involved in ASM contraction; their study will certainly offer knowledge to uncover a potential therapeutic approach for chronic respiratory diseases like asthma. Therefore, we theorized that, in ASM, KCl activates RTKs and their downstream signaling cascades (i.e., MAPK and ROCK) and studied whether these cascades participate in KCl-induced ASM contraction. Because asthmatic airway smooth muscle is slightly depolarized, we employed a low KCl concentration (20 mM) to emulate such a state and examined whether this stimulus could play a role in the activation of receptor tyrosine kinase (RTK) and its downstream signaling pathways. We hypothesized that the characterization of these pathways could help to clarify how ASM contraction response might be altered and, therefore, predispose the tissue to hyperresponsiveness.

## 2. Materials and Methods

### 2.1. Animals

Male Harley guinea pigs weighing between 200 and 300 g were used. They were grown at the animal facility of the Instituto Nacional de Enfermedades Respiratorias under standard controlled conditions: filtered conditioned air, temperature of 21 ± 1 °C, with 50–70% humidity, in a clean bed, with free access to food and water, with a light and dark cycle of 12–12 h.

### 2.2. Organ Bath Experiments

The animals were sacrificed by an overdose of sodium pentobarbital (30 mg/Kg, i.p.) and exsanguinated by an incision of the posterior vena cava. The trachea was immediately obtained, dissected of adipose and connective tissue with a stereoscopic microscope, and eight segments, each containing four cartilage rings, were obtained. Each preparation was suspended in an isolated organ bath chamber containing 5 mL of Krebs Ringer solution with the following composition (mM): 120 NaCl, 4.77 KCl, 1.2 KH_2_PO_4_, 1.2 MgSO_4_, 25 NaHCO_3_, 2 CaCl_2_, and 11 glucose. The solution was continuously bubbled with a mixture of 95% O_2_ and 5% CO_2_ and kept at a pH of 7.4 at 37 °C. Each tissue sample was attached to an isometric tension transducer (Model FT03, Grass Instruments, West Warwick, RI, USA) connected to a programmable signal amplifier–conditioner (CyberAmp 380, Axon Instruments, San Jose, CA, USA), which, in turn, was connected to an analog digital converter (Digidata 1200a, Axon Instruments, San Jose, CA, USA). Isometric tension was recorded and stored for later analysis on a computer, and the data were processed using an Axoscope Version 9.2.0.12 (Axon Instruments 2004). The preparations were maintained at a tension of 1 g for 30 min before starting the experiments. Each tracheal segment was stimulated three times with 60 mM KCl for 30 min until its maximal contraction response was obtained. Afterwards, a 20 mM KCl stimulus lasting 30 min was given. The tissue was then washed 3 times with Krebs solution until a baseline was reached; 30 min later, a second 20 mM KCl stimulus was administered. The protocol described above was followed in other experiments employing 60 mM KCl instead of 20 mM. In this case, tissues received only PD98059 (MEK inhibitor: 5 and 10 μM, *n* = 8 each) 30 min before the second 60 mM stimulus.

In a set of experiments, tissues were incubated 30 min before the second response to 20 mM KCl with genistein (RTK inhibitor: 10 μM, *n* = 10) or ST638 (a selective epidermal growth factor receptor [EGFR] inhibitor [20,21], 10 μM, *n* = 9); FR180204 (ERK 1/2 inhibitor: 5 and 10 μM, *n* = 10 and 32 μM, *n* = 6); SCH772984 (selective ERK1/2 inhibitor, 5, 10 and 32 μM *n* = 6, each); PD98059 (MEK inhibitor: 5 and 10 μM, *n* = 10 each); U0126 (MEK inhibitor: 5 and 10 μM, *n* = 7 each); U0124 (inactive analog of U0126: 5 and 10 μM, *n* = 7 each); Y-27632 (ROCK inhibitor: 5 and 10 μM, *n* = 7 each); or ML-7 (MLCK inhibitor: 5 and 10 μM, *n* = 7 each) (Table 1).

At the end of each experiment, tissues were placed in cryotubes and immediately frozen in liquid nitrogen. These samples were stored at −70 °C until thawed for Western blot analysis.

### 2.3. Western Blot Assays to Define ERK1/2 and MYPT1 Phosphorylation

The frozen tissues obtained from the organ bath experiments were thawed on an ice bed, and pooled: two tissues from the same experimental condition were considered as *n* = 1. Smooth muscle from each sample was carefully dissected and placed in 400 μL of lysis buffer solution (1% Triton, 50 mM Tris at pH 7.4, 150 mM NaCl, 1 mM EDTA, 5 mM NaF, 1 mM sodium pyrophosphate, 1 mM β-glycerophosphate, 2 mM sodium orthovanadate, 1% sodium deoxycholate, 0.1% SDS) and a protease inhibitor cocktail (Protease Inhibitor Cocktail Set III, Calbiochem^®^ Cat. No. 539134, 1:200 *v*/*v*) and finely cut with scissors; afterwards, it was allowed to rest for 30 min at 4 °C. Each sample was then mechanically homogenized (Omni Tip Plastic Homogenizer Probes, OMNI International, USA, Kennesaw, GA) and centrifuged at 2000 rpm for 5 min at 4 °C; the supernatant was then recovered using a micropipette. Subsequently, the supernatant was concentrated by centrifugation at 3000 rpm for 20 min at 4 °C using an Amicon ^®^ Ultra-4 membrane (Cork, Ireland) (30,000 Molecular Weight Cutoff; Millipore Cat. No.: UFC803096). The protein determination was carried out by the bicinchoninic acid (BCA) method using a commercial kit (Pierce^TM^ BCA Protein Assay Kit, Thermo Fisher Scientific Mexico, Mexico City, Mexico, Cat. No. 23225).

To separate the proteins through electrophoresis, 10 μg of protein per sample were used. Then, 6 μL of loading solution (2X Laemmli Sample Buffer, Bio-Rad, Mexico City, Mexico, Cat No.: 1610737) was added to each sample and then heated at 100 °C for 5 min in a water bath. Subsequently, protein separation was carried out using 8% sodium dodecyl sulfate polyacrylamide gels with a buffer solution (0.025 M Tris, 0.192 M glycine and 0.1% SDS) for 2 h at 100 V at 4 °C. The proteins were transferred to a nitrocellulose membrane with a transfer buffer (0.025 M Tris, 0.192 M glycine, and 20% methanol) for 1 h at 100 V at 4 °C. Afterwards, each membrane was blocked using 7% non-fat dry milk reconstituted in a saline–Tris buffer containing 0.5% Tween-20 (TBS-T) for 1 h at room temperature. The membranes were washed with TBS-T and incubated overnight at 4 °C with the corresponding rabbit polyclonal antibodies (primary antibodies) listed in Table 2.

The next day, the membranes were washed with TBS-T and incubated with the secondary anti-rabbit antibody conjugated with peroxidase (HRP) that allowed the detection of the primary antibody (Anti-rabbit IgG HRP; 1:2000 *v*/*v*, Merck Millipore, Rahway, NJ, USA). Protein detection was performed using a chemiluminescent substrate (Super Signal West Femto Maximum Sensitivity Substrate, cat. 34096; Thermo Scientific, Rockford, IL, USA) through in Molecular Imaging ChemiDoc XRS+, Model Universal Hood, Hercules, CA, USA.

### 2.4. Statistical Analysis

For all experimental results obtained in isolated organ baths, tension measurements were evaluated as a percentage of the first control response to 20 mM KCl. To assess organ bath and densitometric results, we employed a software (inerSTAT-a v1.7 b) to run a one-way ANOVA test followed by a Dunnett’s ad hoc test. Significant differences were considered when * *p* < 0.05 or ** *p* < 0.01. Data expressed in the figures and texts are mean ± standard error of the mean; for organ bath experiments, “*n*” represents the number of animals used. In densitometry, each “*n*” corresponds to 3 separated WBs.

### 2.5. Densitometry Analysis

The relative levels of ERK1/2 and MYPT1 phosphorylation were normalized to total ERK and total MYPT1, respectively. Data are expressed as a percentage of the control response (20 mM and 60 mM KCl) and are represented as the mean ± standard error of the mean (*n* = 3). The “*n*” corresponds to 3 separated WBs. In most cases, an ad hoc Dunnett’s test was used to analyze the differences in ERK and MYPT1 expression during contraction induced by 20 mM KCl. Only ERK phosphorylation was analyzed for the SCH772984 results following the same methodology. In the case of the experiment of 60 mM KCl and PD98059 (5 and 10 μM), ERK1/2 phosphorylation was normalized to total ERK. * *p* < 0.05 and ** *p* < 0.01 were considered significant.

## 3. Results

**Figure 1:**
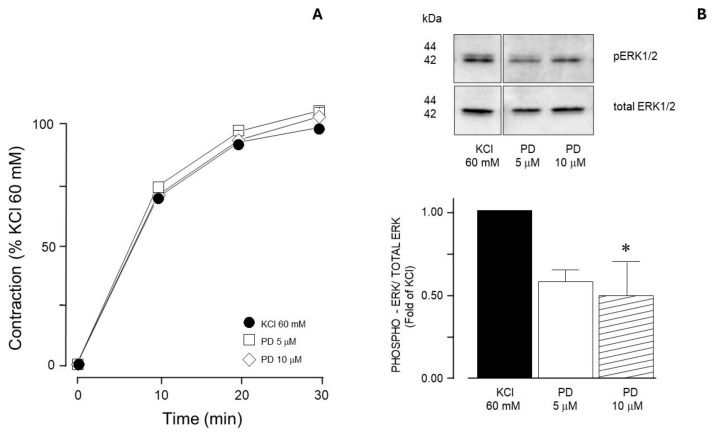
MEK inhibition does not modify 60 mM KCl-induced contraction in guinea pig airway smooth muscle. (**A**) PD98059 (MEK inhibitor; 5 and 10 μM, *n* = 8, each) did not modify the contraction induced by 60 mM KCl in guinea pig tracheas. Symbols represent mean ± standard error of the mean. (**B**) Upper panel shows a representative Western blot (WB) for 60 mM KCl-induced phosphorylation of ERK1/2 and its modification by PD98059 (PD, 5 μM, and 10 μM). Below, densitometric analysis of ERK1/2 phosphorylation induced by 60 mM KCl. Bar graph showing mean ± standard error of the mean of 3 WBs. The differences found were statistically significant (* *p* < 0.05).

In guinea pig trachea, 60 mM KCl-induced contraction is not diminished by PD98059 (MEK inhibitor; Figure 1A), even though the KCl-induced ERK phosphorylation is significantly lowered (Figure 1B).

**Figure 2:**
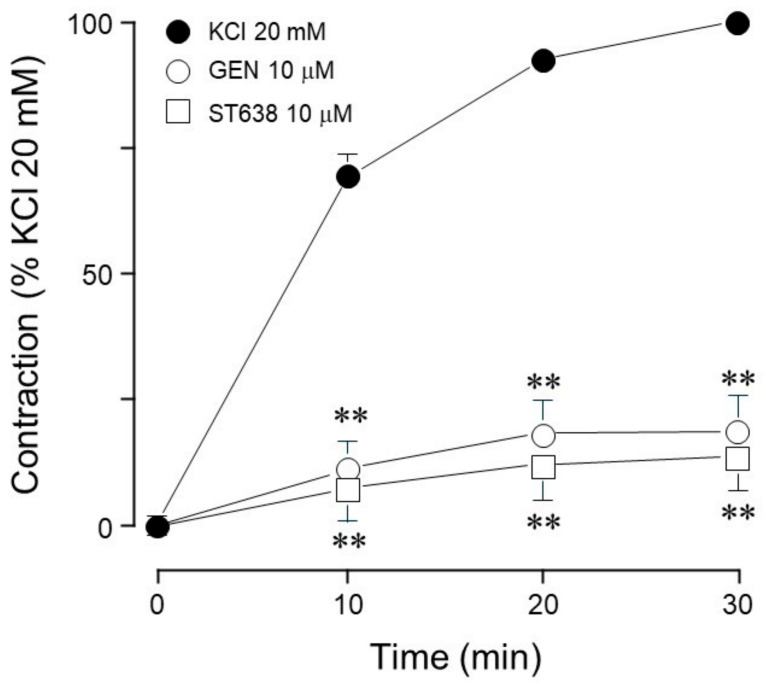
RTK inhibitors diminish the contraction response induced by 20 mM KCl. Genistein (GEN) and ST638 (10 μM, *n* = 10 and *n* = 9, respectively) significantly diminished the contraction induced by 20 mM KCl in guinea pig tracheas. Symbols represent mean ± standard error of the mean. ** *p* < 0.01.

Meanwhile, a 20 mM KCl stimulus includes the participation of RTKs, as can be seen in Figure 2. The RTK inhibitors genistein and ST638, a selective EGFR inhibitor, significantly diminished the contraction. Both inhibitors were used at 10 μM, a concentration in accordance with former reports [14,22].

**Figure 3:**
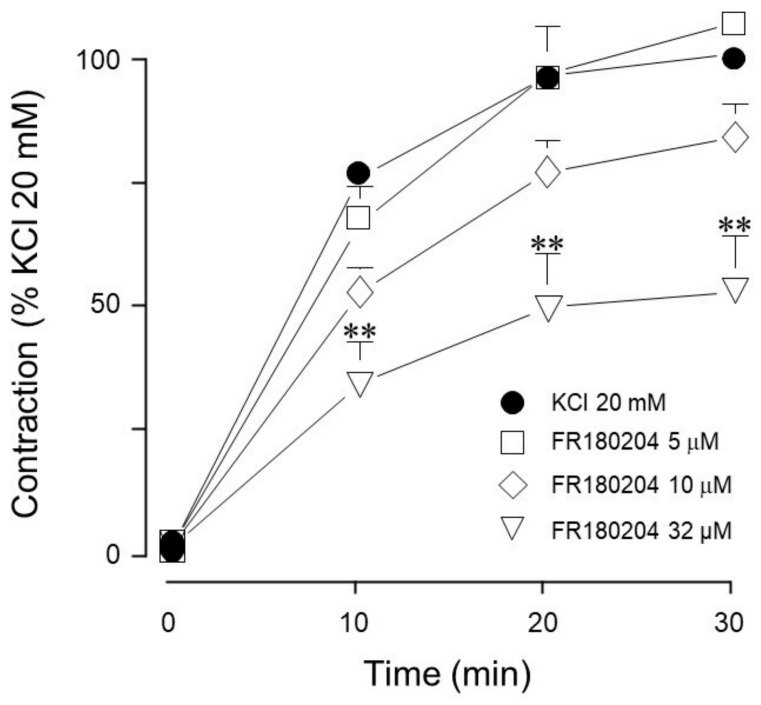
Effect of an ERK inhibitor on the contraction response induced by 20 mM KCl. Only at the highest concentration tested (32 µM, *n* = 6) did FR180204 inhibit the contraction induced by 20 mM KCl in guinea pig trachea, while lower concentrations (5 µM, 10 µM, *n* = 10 each) showed no effect. Symbols represent mean ± standard error of the mean, ** *p* < 0.01.

Because RTKs are coupled to the MEK-ERK signaling pathway, we further explored whether KCl-induced contraction was dependent on these kinases. Our results indicated that, in guinea pig trachea, KCl-induced contraction depends on ERK, since it was diminished by pharmacological inhibition with 32 µM FR180204 (Figure 3).

**Figure 4:**
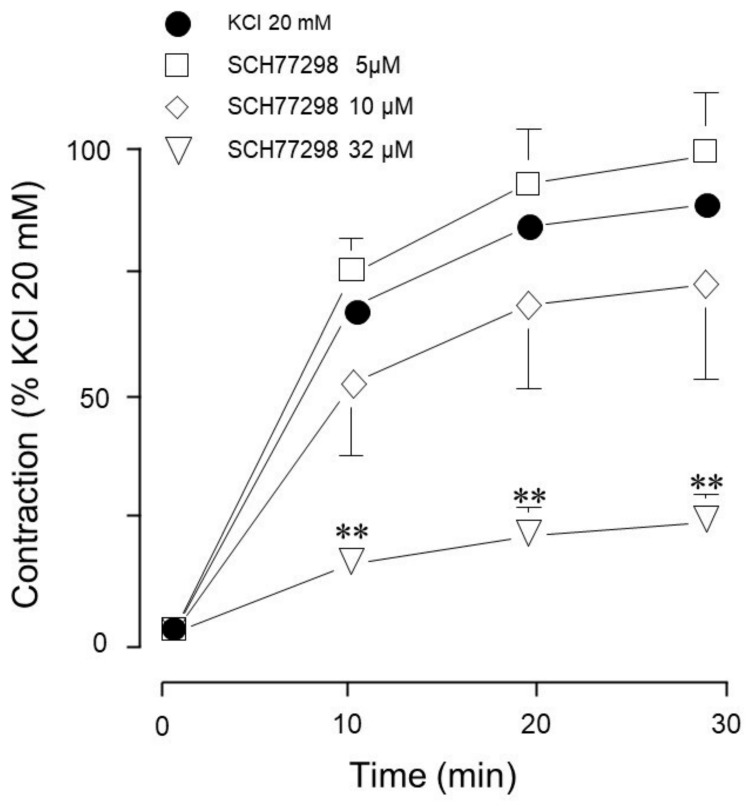
Effect of a selective ERK inhibitor on the contraction induced by 20 mM KCl in guinea pig airway preparations. The KCl-induced contraction was inhibited by SCH772984 (SCH77298), 32 µM (*n* = 6). Lower concentrations (5 µM, 10 µM, *n* = 6 each) showed no effect. Symbols represent mean ± standard error of the mean, ** *p* < 0.01.

Also at 32 µM, SCH772984, a selective ERK 1/2 inhibitor [23], significantly inhibited the 20 mM KCl-induced contraction (Figure 4).

**Figure 5:**
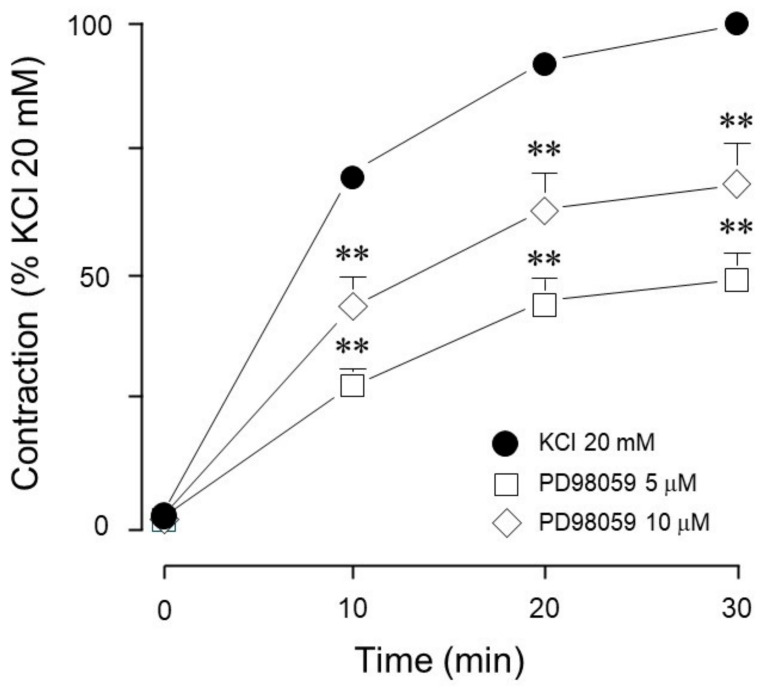
MEK inhibition reduced the contraction induced by 20 mM KCl. PD98059 (5 µM, 10 µM, *n* = 10 each) diminished the contraction induced by 20 mM KCl in guinea pig trachea. Symbols represent mean ± standard error of the mean, ** *p* < 0.01.

To further explore whether MEK could be involved in this phenomenon, we employed PD98059 (a MEK inhibitor) and appreciated a significant decrease of the KCl-induced contraction (Figure 5).

**Figure 6:**
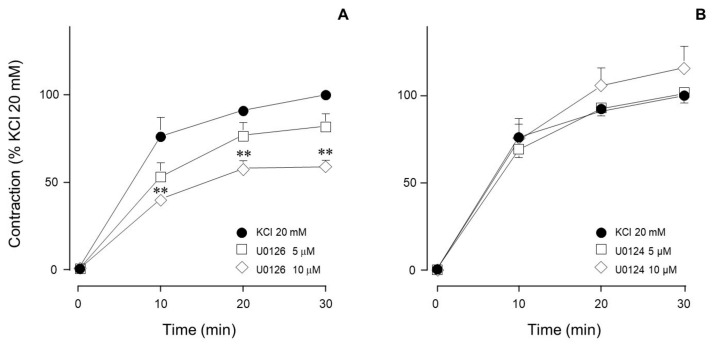
MEK inhibition reduced the contraction induced by 20 mM KCl. (**A**) U0126 (5 µM, 10 µM, *n* = 7 each) diminished the contraction induced by 20 mM KCl in guinea pig trachea. (**B**) An inactive analog of U0126 (U0124, 5 µM, 10 µM, *n* = 7 each) had no effect on the contraction. Symbols represent mean ± standard error of the mean, ** *p* < 0.01.

This result was further corroborated by another set of experiments in which we used U0126, another MEK inhibitor, and its inactive analog (U0124). Our results confirmed the participation of MEK in the KCl-induced contraction, since its inhibition through U0126 significantly reduced the contraction (Figure 6A), while U0124 had no effect (Figure 6B).

**Figure 7:**
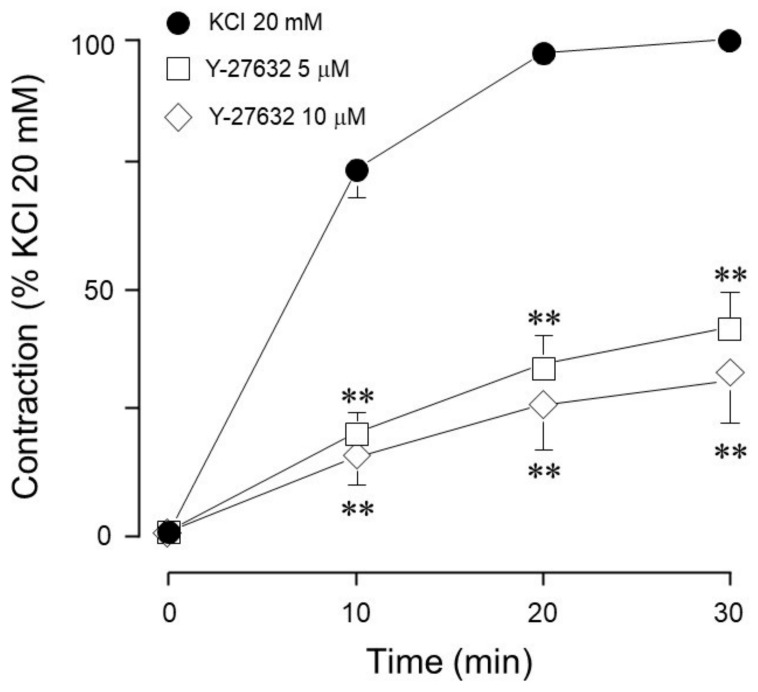
ROCK inhibition reduces the contraction induced by 20 mM KCl. Y-27632 (5 µM, 10 µM, *n* = 7 each) significantly decreased the 20 mM KCl-induced contraction in guinea pig trachea. Symbols represent mean ± standard error of the mean, ** *p* < 0.01.

We also demonstrated that, in guinea pig trachea, ROCK plays a role in KCl-induced contraction, because Y-27632 (a ROCK inhibitor) significantly decreased the 20 mM KCl-induced contraction (Figure 7).

**Figure 8:**
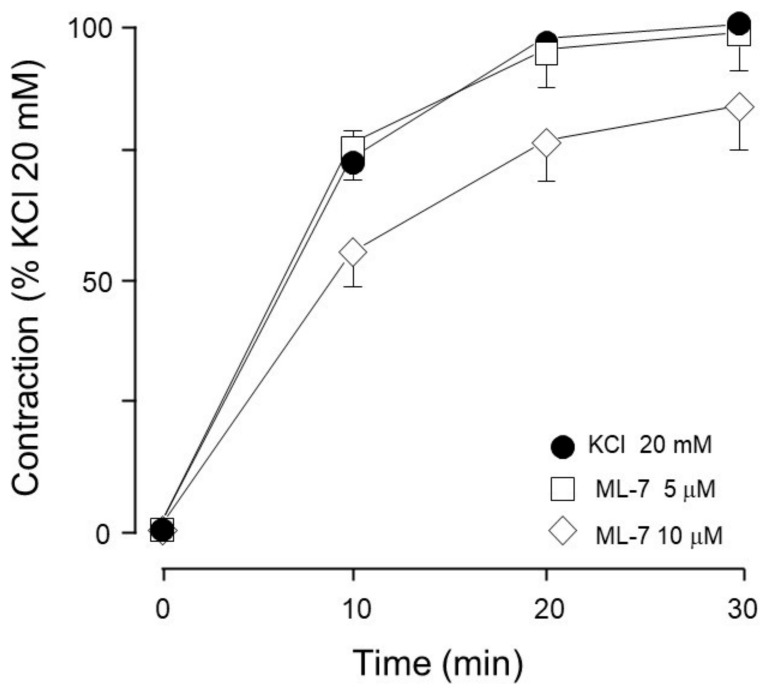
MLCK inhibition did not modify the 20 mM KCl-induced contraction. ML-7 (5 µM, 10 µM, *n* = 7 each) had no effect on the contraction induced by 20 mM KCl in guinea pig trachea.

Meanwhile, MLCK inhibition did not modify the KCl-induced contraction (Figure 8).

**Figure 9:**
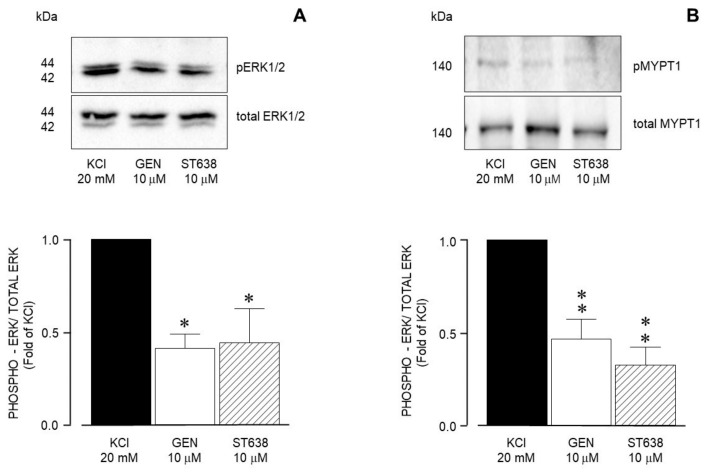
Densitometric analysis to define RTK participation in the 20 mM KCl-induced phosphorylation of ERK1/2 and MYPT1 in guinea pig airway smooth muscle. (**A**) Upper panel shows a representative Western blot (WB) for the 20 mM KCl-induced phosphorylation of ERK1/2 and its modification by genistein (GEN, 10 μM) and ST638 (10 μM). Below, bar graph showing mean ± standard error of the mean of 3 WBs. The differences found were statistically significant (* *p* < 0.05). (**B**) Upper panel shows a representative Western blot (WB) for the 20 mM KCl-induced phosphorylation of MYPT1 and its modification by genistein (GEN, 10 μM) and ST638 (10 μM). Below, bar graph showing mean ± standard error of the mean of 3 WBs. The differences found were statistically significant (** *p* < 0.01).

In our Western blot experiments, we observed that the addition of 20 mM KCl to the airway smooth muscle organ bath preparations induced the phosphorylation of both ERK and MYPT1 (Supplementary Figure S1). Other results showed that RTK inhibition by genistein and ST638 significantly diminished ERK phosphorylation (Figure 9A) and MYPT1 phosphorylation (Figure 9B) in accordance with the findings illustrated in Figure 2.

**Figure 10:**
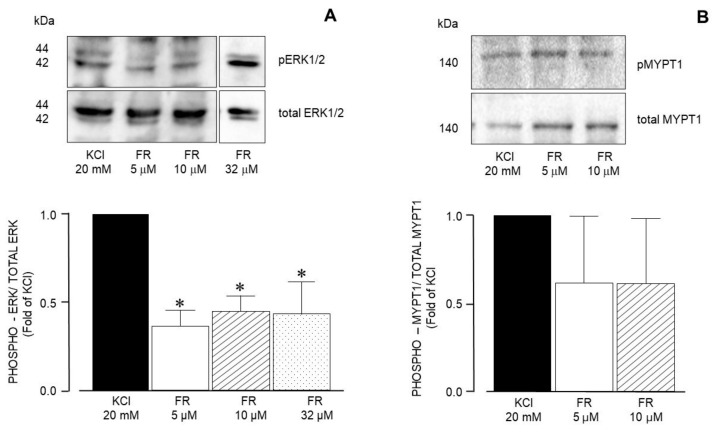
Densitometric analysis to define ERK1/2 and MYPT1 phosphorylation induced by 20 mM KCl in guinea pig airway smooth muscle. (**A**) Upper panel shows a representative Western blot (WB) for the 20 mM KCl-induced phosphorylation of ERK1/2 and its modification by FR180204 (FR, 5 μM, 10 μM, and 32 μM). Below, bar graph showing mean ± standard error of the mean of 3 WBs. The differences found were statistically significant (* *p* < 0.05). (**B**) Upper panel shows a representative Western blot (WB) for the 20 mM KCl-induced phosphorylation of MYPT1 and its modification by FR180204 (FR, 5 μM and 10 μM). Below, bar graph showing mean ± standard error of the mean of 3 WBs.

ERK’s phosphorylation induced by 20 mM KCl was significantly diminished by FR180204 at all concentrations used (Figure 10A), while MYPT1 was not altered by this inhibitor (Figure 10B).

**Figure 11:**
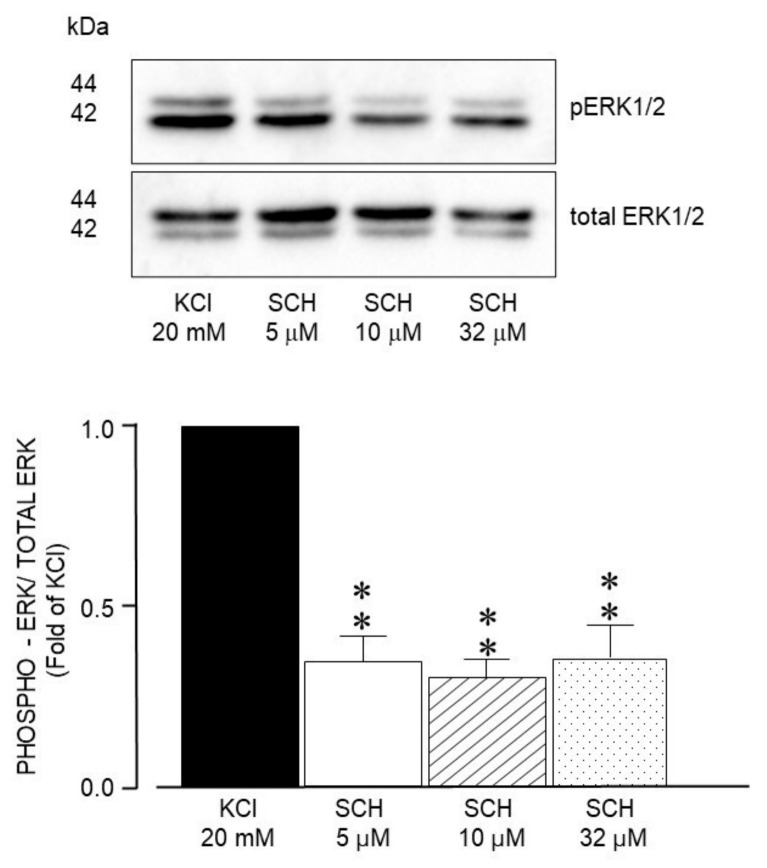
Densitometric analysis to define ERK1/2 phosphorylation induced by 20 mM KCl in guinea pig airway smooth muscle. Upper panel shows the modification of the ERK 1/2 phosphorylation state induced by SCH772984 (SCH, 5 μM, 10 μM, and 32 μM). Bar graph depicts significant inhibition of ERK phosphorylation (** *p* < 0.01). Mean ± standard error of the mean of 3 Western blots (WBs) are represented in each bar.

The selective ERK 1/2 inhibitor SCH772984 also diminished ASM phosphorylation at all concentrations tested (Figure 11).

**Figure 12:**
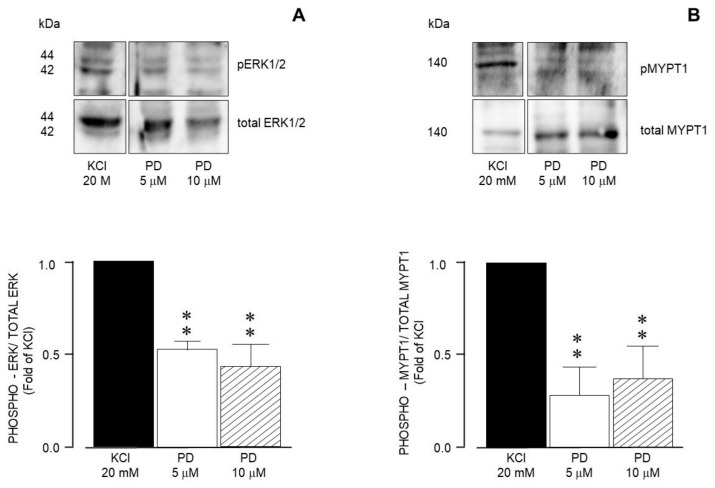
Densitometric analysis to define MEK participation in the ERK1/2 and MYPT1 phosphorylation induced by 20 mM KCl in guinea pig airway smooth muscle. (**A**) Upper panel shows a representative Western blot (WB) for the 20 mM KCl-induced phosphorylation of ERK1/2 and its modification by PD98059 (PD, 5 μM, and 10 μM). Below, bar graph showing mean ± standard error of the mean of 3 WBs. The differences found were statistically significant (** *p* < 0.01). (**B**) Upper panel shows a representative Western blot (WB) for the 20 mM KCl-induced phosphorylation of MYPT1 and its modification by PD98059 (PD, 5 μM, and 10 μM). Below, bar graph showing mean ± standard error of the mean of 3 WBs. The differences found were statistically significant (** *p* < 0.01).

Meanwhile, MEK inhibition with PD98059 decreased both ERK phosphorylation (Figure 12A) and MYPT1 phosphorylation (Figure 12B) induced by 20 mM KCl. This result is congruent with the effect shown by PD98059 on the KCl-induced contraction depicted in Figure 5.

**Figure 13:**
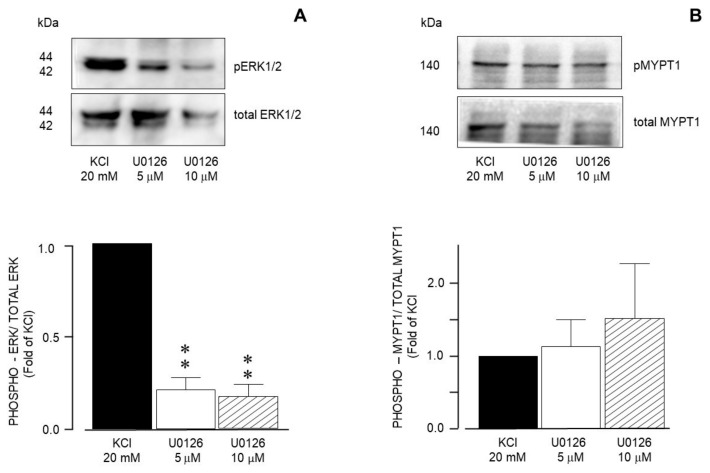
Densitometric analysis to define MEK participation in the ERK1/2 and MYPT1 phosphorylation induced by 20 mM KCl in guinea pig airway smooth muscle. (**A**) Upper panel shows a representative Western blot (WB) for the 20 mM KCl-induced phosphorylation of ERK1/2 and its modification by U0126 (5 μM and 10 μM). Below, bar graph showing mean ± standard error of the mean of 3 WBs. The differences found were statistically significant (** *p* < 0.01). (**B**) Upper panel shows a representative Western blot (WB) for the 20 mM KCl-induced phosphorylation of MYPT1 and its modification by U0126 (5 μM and 10 μM). Below, bar graph showing mean ± standard error of the mean of 3 WBs. Note that (**A**,**B**) have different scales on the Y axis.

**Figure 14:**
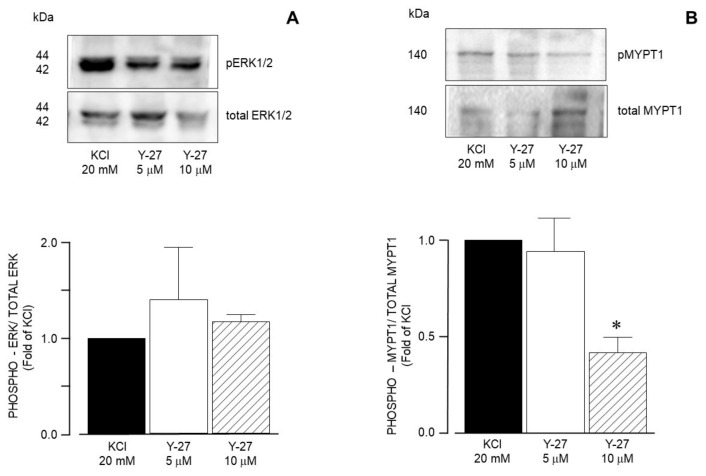
Densitometric analysis to define ERK1/2 and MYPT1 phosphorylation induced by 20 mM KCl in guinea pig airway smooth muscle. (**A**) Upper panel shows a representative Western blot (WB) for the 20 mM KCl-induced phosphorylation of ERK1/2 and its modification by Y27632 (Y-27, 5 μM, and 10 μM). Below, bar graph showing mean ± standard error of the mean of 3 WBs. (**B**) Upper panel shows a representative Western blot (WB) for the 20 mM KCl-induced phosphorylation of MYPT1 and its modification by Y27632 (Y-27, 5 μM, and 10 μM). Below, bar graph showing mean ± standard error of the mean of 3 WBs. The difference found was statistically significant (* *p* < 0.05). Note that (**A**,**B**) have different scales on the Y axis.

Furthermore, the use of U0126, a specific MEK inhibitor, significantly reduced the ERK phosphorylation induced by 20 mM KCl (Figure 13A), but did not alter MYPT1 phosphorylation (Figure 13B). Both U0126 concentrations tested (5 and 10 μM) reduced ERK phosphorylation, but only the highest concentration lowered the KCl-induced contraction (Figure 6A). Finally, the addition of Y-27632 (a ROCK inhibitor) did not modify KCl-induced ERK phosphorylation (Figure 14A), but significantly diminished MYPT1 phosphorylation induced by the KCl addition (Figure 14B). This result is in accordance with the effect produced by Y-27632 in the organ bath experiments (Figure 7).

## 4. Discussion

For many years, a compelling question has persisted: is KCl-induced airway smooth muscle contraction Ca^2+^-dependent or sensitization-driven? In this regard, our group has developed this research with the intent of answering this question. In studies carried out in bovine tracheal smooth muscle, we frequently observed that KCl-induced contraction was not completely abolished by the addition of nifedipine [4]. This residual contraction (about 20% of the initial KCl-induced contraction) was abated by the addition of Y-27632 (a ROCK inhibitor). Because this nifedipine-resistant, ROCK-dependent contraction seems to be Ca^2+^-independent, we studied KCl (60 mM)-induced contraction in a Ca^2+^-free medium and found that Na^+^ plays an important role in this phenomenon [24]. Na^+^ handling mechanisms involved in airway smooth muscle contraction are complex and have been scantly studied [25]; notwithstanding, Na^+^ is the main ion involved in membrane potential maintenance and in initiating membrane depolarization in response to agonist stimulation. In summary, the airway smooth muscle contraction induced by KCl is constituted by a Ca^2+^-dependent component (nifedipine-sensitive) and a Ca^2+^-independent (sensitization-driven, ROCK-dependent) component. We hypothesized that a lower KCl concentration (20 mM) would induce the activation of the sensitization-driven component and therefore proceeded to develop the experiments described herein. Interestingly, when comparing the MEK inhibitor’s effect on the contraction induced by 60 mM KCl vs. those caused by 20 mM KCl, we observed that the former induced no alteration in the contraction (Figure 1), while the latter was significantly diminished. This finding suggests that MEK activation (through RTKs) might depend on the membrane voltage that, with a 20 mM KCl stimulus, theoretically ought to be lower than the opening threshold for the L-type voltage-dependent Ca^2+^ channels. Most probably, under our experimental conditions, MEK’s participation in KCl contraction does not depend on intracellular Ca^2+^ augmentation; i.e., it is a Ca^2+^-independent phenomenon, as we have demonstrated for ROCK. Furthermore, in this tissue, ROCK activation seems to develop independently of the KCl concentration employed, since both 60 mM [4] and 20 mM (herein, Figure 14) induced MYPT1 phosphorylation.

For many years, KCl was considered a trustworthy pharmacological tool to study ASM contraction bypassing G protein-coupled receptor activation and the well-known second messenger signaling cascade. However, there is evidence to indicate that in this tissue, KCl-induced contraction can include the activation of ROCK and other kinases [4,5,26,27,28]. Furthermore, ASM stimulation with different KCl concentrations seemingly results in the activation of different signaling pathways; higher (60 mM) concentrations activate ROCK and MEK (Figure 1), but only the former participates in the contraction. Meanwhile, with a 20 mM stimulus, both ROCK and MEK are activated and participate in the contraction. In asthmatic airways, ASM is slightly depolarized [29], a circumstance that could predispose the tissue to airway hyperresponsiveness, a hallmark of this ailment that might be related to the continuous activation of certain signaling pathways. In this sense, the sustained stimulation of RTKs could contribute to ASM hypertrophy, another characteristic of the asthmatic airway. To emulate such a depolarized state, we used a low KCl concentration (20 mM) and, through a pharmacological approach, investigated whether receptor tyrosine kinase (RTK) participates in the airway smooth muscle contraction induced by this substance. To achieve this, we employed ST638, a selective EGFR inhibitor, and genistein that has been cataloged as a multimodal drug because it promotes apoptosis and cell cycle arrest, inhibiting metastasis. Moreover, it has been demonstrated to be an excellent antioxidant and anti-inflammatory agent [30]. In the present study, both agents were employed as RTK inhibitors under the same experimental conditions to improve the results’ certainty. We found that RTK inhibition significantly diminished KCl-induced contraction (Figure 2), in agreement with former studies carried out in vascular smooth muscle [10] and bronchial smooth muscle [14]. Furthermore, we observed that KCl induces ERK phosphorylation in ASM (Figure 9A, Figure 10A, Figure 11, Figure 12A, Figure 13A, and Figure 14A), and that this phenomenon was diminished by RTK inhibition (Figure 9A). In this sense, abundant reports consistently support the fact that RTK activation unleashes the Ras/MAPK/ERK signaling pathway [16,31,32,33], mostly via synthetic signaling.

On the other hand, KCl-induced smooth muscle contraction implies the opening of L-type (long-lasting) voltage-dependent Ca^2+^ channels (L-VDCC) [34,35,36]; in turn, Ca^2+^ entry through L-VDCC is followed by the formation of the Ca^2+^/calmodulin (CaM) complex that may directly activate the Ras/MAPK/ERK signaling cascade [37]. It has been shown that CaMK (Ca^2+^/calmodulin kinase) regulates depolarization-induced MEK-ERK activity [38,39]. Nonetheless, this signaling pathway is mostly activated through RTK stimulation, whether by its cognate agonist or, seemingly, by membrane depolarization. This stimulation activates the GTP-binding protein Ras, which binds and activates Raf (mitogen-activated protein kinase kinase), which phosphorylates and activates MEK, responsible for phosphorylating and activating MAPK/ERK [40].

It has been observed that L-VDCC inhibition interrupts the generation of immediate early genes (IEGs) [41]. In fact, its activation induces IEGs in vascular smooth muscle [42], human ASM [43,44], and airway epithelia [45]. IEGs cipher for a variety of cytoplasmic enzymes, secreted proteins, and transcription factors related to cell differentiation, metabolism, and proliferation. They are typically stimulated by an extracellular signal (e.g., growth factors, mitogens, ultra violet light [UV], toxins) [46,47] and their expression is rapid and transient [48]. For instance, the peak expression of FOS is 30 to 60 min after stimulation [49]. As they do not require protein synthesis, translational inhibitors have no effect on their expression [48,50], which is known to be very low, partially due to mRNA instability and to efficient proteolytic degradation [51].

**Figure 15:**
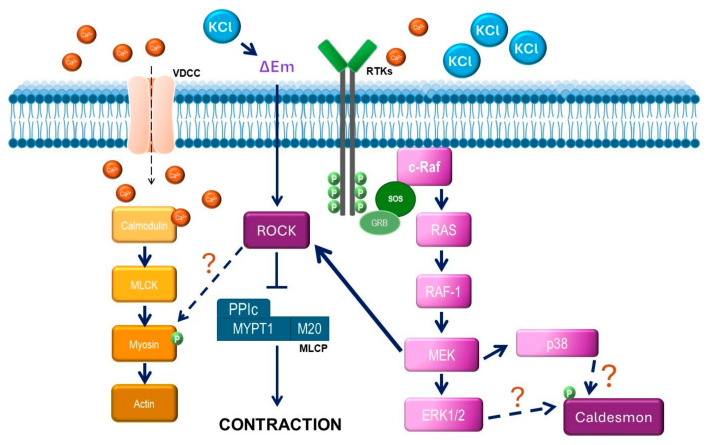
Schematic representation of the effects of KCl on airway smooth muscle. KCl favors membrane depolarization (ΔEm). This depolarization might induce ROCK activation that, in turn, inhibits myosin light chain phosphatase (MLCP) by phosphorylating its myosin-targeting subunit (MYPT1). On the other hand, ROCK may phosphorylate myosin directly. Both sensitization (Ca^2+^-independent) mechanisms contribute to airway contraction. KCl-induced membrane depolarization (ΔEm) also induces RTKs (most probably EGFR) to activate MEK, which phosphorylates ERK 1/2 and could directly phosphorylate ROCK and p38. Because ERK1/2 pharmacological inhibition alters KCl-induced contraction, this kinase may phosphorylate caldesmon under this experimental condition. Whether KCl-activated MEK induces caldesmon phosphorylation through p38 MAPK or through ERK 1/2 in airway smooth muscle remains an open question that deserves further study (marked by the “?” symbols).

Conceivably, ASM depolarization with KCl activates the Ras/MAPK/ERK signaling cascade through RTK stimulation. To elucidate whether ERK participates in KCl-induced contraction, we used FR180204, an ERK inhibitor. As illustrated in Figure 3, only at a high concentration did this inhibitor significantly diminish the KCl-induced contraction. To further confirm this finding, we employed another ERK 1/2 inhibitor (SCH772984) and observed similar results: only the highest concentration tested inhibited KCl-induced contraction, confirming that ERK 1/2 participates in this phenomenon (Figure 4 and Figure 11). To further explore KCl-induced signaling pathways, we employed PD98059, a potent and selective MEK inhibitor [52], which significantly decreased KCl-induced contraction (Figure 5) and ERK phosphorylation (Figure 12A). Since the primary kinase upstream of ERK is MEK, the latter likely participates in KCl-induced contraction in ASM. Furthermore, this finding was corroborated by another set of experiments using U0126 (another MEK inhibitor) and U0124 (a useful negative control for MEK inhibitors). Again, the results shown in Figure 6A demonstrate that MEK’s inhibition diminished KCl-induced contraction, while the inhibitor’s negative control had no effect (Figure 6B). Meanwhile, ERK phosphorylation was also reduced by U0126 (Figure 13A). Figure 10A and Figure 11 illustrate that ERK is phosphorylated by the 20 mM KCl stimulus, and this phosphorylation is diminished by the pharmacological inhibitors FR 180204 and SCH772984. Its phosphorylation was also significantly diminished by MEK inhibitors (PD98059, Figure 12A and U0126, Figure 13A). While MEK inhibition significantly reduced contraction, only the highest FR 180204 and SCH772984 concentration (both at 32 μM) showed the same effect. These results confirm the participation of ERK 1/2 in the ASM contraction induced by 20 mM KCl. Notwithstanding, MEK may also influence contraction through ERK-independent pathways, such as cross-talk with other kinases (e.g., p38 MAPK, JNK) or cytoskeletal regulators. Under our experimental conditions (KCl stimulus), ERK might play a role in the phosphorylation of caldesmon, contributing to ASM-sustained contraction. In airway smooth muscle, caldesmon, a calmodulin-binding protein that inhibits myosin ATPase activity, is phosphorylated by MAP kinases and, in turn, controls or modulates smooth muscle contraction in concert with myosin light chain phosphorylation. It has been established that, in ASM, a variety of contractile agonists induce ERK and p38 MAP kinase family members to phosphorylate caldesmon [53,54,55]. Whether the KCl-activated MEK-ERK pathway induces caldesmon phosphorylation in airway smooth muscle remains an open question that deserves further study (Figure 15).

We also observed that, in KCl-stimulated guinea pig trachea, RTK inhibition diminished myosin phosphatase targeting subunit (MYPT1) phosphorylation (Figure 9B), a phenomenon closely related to the decrease in the tissues’ contraction seen during the same proceeding (Figure 2). In this sense, ROCK-phosphorylated MYPT1 participation in smooth muscle contraction has been reported before. In ASM, the inhibition of the myosin light chain phosphatase is a key contributor to the sustained contraction phenomenon. This phosphatase is constituted by a catalytic subunit; a targeting, myosin-binding subunit (MYPT1); and a third subunit of unclear purpose. It has been established that MYPT1 phosphorylation by Rho kinase (ROCK) at T696 and T853 inhibits phosphatase activity [56].

Interestingly, a previous study carried out in bovine tracheal ASM reports that KCl stimulation induced T853 and T696 phosphorylation, and that the latter is preferentially phosphorylated during sustained contraction [57]. In comparison to these authors who used 75 mM KCl, in the present work, we used only 20 mM KCl in guinea pig ASM and observed MYPT1 KCl-induced phosphorylation (Figure 9B, Figure 10B, Figure 12B, Figure 13B and Figure 14B; Supplementary Figure S2). By pharmacologically inhibiting RTKs with genistein and ST638, we observed a decrease in KCl-induced MYPT1 phosphorylation in T696 (Figure 9B), a finding that could be related to the lesser contractile response observed in the organ bath experiments (Figure 2) when following the same experimental protocol.

On the other hand, MYPT1 phosphorylation was not modified by the ERK inhibitor FR180204 (Figure 10B), but was significantly diminished by the addition of PD98059, a MEK inhibitor (Figure 12B), and not by U0126, another MEK inhibitor (Figure 13B). In this regard, it has been reported that U0126 possesses greater potency than PD98059 [55], a fact that was also noticed in our experimental results for KCl-induced ERK phosphorylation. When comparing PD98059 effects on ERK phosphorylation (Figure 12A) against U0126 (Figure 13A), the inhibition induced by the latter is about 25% higher, illustrating its greater effect (note that both inhibitors were tested at 5 and 10 µM). Regarding U0126’s inhibitory effects on KCl-induced MYPT1 phosphorylation, it did not modify it, indicating that, when MEK is efficiently inhibited, its effect on MYPT1 is null. As already mentioned, in ASM, MYPT1 phosphorylation is primordially carried out by ROCK; in this sense, when we employed a ROCK inhibitor (Y-27632), we observed a significant reduction in KCl-induced contraction (Figure 7) and MYPT1 phosphorylation (Figure 14B). This inhibitor had no effect on the ERK phosphorylation induced by KCl (Figure 14A). Surprisingly, the inhibition of the myosin light chain kinase (MLCK) with ML-7 did not modify KCl-induced contraction in ASM (Figure 8). In this sense, Saponara et al. [58] found, in rat vascular tissues, that ML-7 effects include the inhibition of L-VDCC activity in a concentration-dependent manner and, moreover, its effect is voltage-dependent (its efficacy increased at more depolarized holding potential). Conceivably, the depolarization generated by the addition of 20 mM KCl in ASM was not high enough to generate a proper ML-7 inhibition of the MLCK. A more straightforward interpretation could be that MLCK is not the main kinase involved in this process. Instead, because the inhibitor Y-27632 suppressed the effect (Figure 7 and Figure 14B), it further indicates that the ROCK pathway is a key mediator in this response. This result is not new, since our group’s former research in bovine airway smooth muscle showed that KCl-induced contraction is significantly diminished by Y-27632 [4]. Worthy of mention are the different experimental conditions in which that protocol and the one described herein were carried out. In the present study, tissues were stimulated with 20 mM KCl, and in the former, we used 60 mM KCl. Notwithstanding, both contractions were significantly diminished by Y-27632 (10 µM), suggesting that ROCK-dependent contraction in airway smooth muscle mainly relies on membrane depolarization rather than in intracellular Ca^2+^ concentration increment (Figure 13). Interestingly, the mechanism whereby the Rho-ROCK pathway is activated upon depolarization has not been completely understood and implies that Rho GTPases are sensitive to physical stimuli. This assumption might guide multiple theories in the study of asthmatic airways since considerable knowledge about hyperresponsiveness and deep inspiration-induced relaxation have been gathered in contrast to the relatively small amount of knowledge regarding the activation and regulation of their signaling processes. ROCK has been shown to phosphorylate MLC directly [59,60,61] on Ser19 and Thr18, but this effect might have less physiological significance in healthy smooth muscle. Indeed, research carried out in vascular [62] and colon [63] smooth muscles demonstrates ROCK’s capability to phosphorylate MLC and induce contraction in these tissues, but evidence in airway smooth muscle is scant. In accordance with our results, we consider that the physiological importance of the depolarization-induced ROCK activation and its actions on MLC warrant future studies to further explore asthma-related airway smooth muscle malfunctions like hyperresponsiveness and altered deep inspiration-induced relaxation.

In summary, in guinea pig ASM, KCl stimulation activates MEK and ROCK through RTKs and, therefore, the triggering mechanisms of these kinases might be connected. Indeed, both signaling pathways include an upstream small GTPase: Ras in the case of MEK and Rho for ROCK. Ras and Rho are classified as small GTPases, molecular switches that are inactive while bound to GDP and active in their GTP-bound state. In this sense, the Ras superfamily of small GTPases includes Ras, Rho, Ran, Rab, and Arf. Each family is further subdivided into subfamilies with a shared G domain that confers them GTPase and nucleotide exchange capacities [64] as, for instance, the Rho family of GTPases, that includes Rac, Rho, and Cdc42 [65]. Small GTPases are located in the plasma membrane, where they transit from the GDP-bound to the GTP-bound state as a consequence of the stimulation of cell surface receptors, as for instance, RTKs [13]. This transition from GDP to the GTP-bound state is aided by guanine nucleotide exchange factors (GEFs), while GTPase-activating proteins (GAPs) convert GTP-bound small GTPase back to the GDP-bound state [66,67].

It has been recognized that Ras and Rho GTPases share some important convergent cellular responses like gene expression, cellular proliferation, and actin cytoskeleton regulation. Interestingly, it has been reported that, in ASM, RhoA-activated ROCK inhibits MLC phosphatase, contributing to this tissue’s sustained contraction [3]. However, it also regulates F-actin dynamics and actin polymerization by activating the serine–threonine kinase Pak, which, in turn, mediates the activation of Cdc42 and neuronal Wiskott–Aldrich syndrome protein (N-WASp) that participate in the nucleation of actin filaments [68,69]. In fact, the cytoskeleton’s dynamics play a preponderant role in ASM contraction. In this sense, it is known that prompt actin polymerization is a response to growth factors like platelet-derived growth factor and that the small GTPase Rac intervenes in the plasma membrane [70], while Rho modulates focal adhesion assembly and actin stress fiber formation [71].

## 5. Conclusions

In ASM, RTK stimulation with a low KCl concentration contributes to the phosphorylation of ROCK, MEK, and ERK1/2, which intervene in the contraction. ROCK is the main kinase responsible for the contraction, while MEK phosphorylates ERK 1/2 and probably p38 that, in turn, could activate caldesmon and induce sustained contraction. These findings indicate that in asthmatic, slightly depolarized ASM, both ROCK and MEK signaling pathways are primed and will contribute to airway hyperresponsiveness. These findings may help to uncover potential therapeutic approaches and therefore deserve further and more accurate research.

## Figures and Tables

**Table 1:** Inhibitors.

Inhibitor Name	Target Molecule	Concentration (µM)
Genistein	RTK	10
ST638	RTK	10
FR180204	ERK1/ERK2	5, 10, 32
SCH772984	ERK1/ERK2	5, 10, 32
PD98059	MEK	5, 10
U0126	MEK	5, 10
U0124	Inactive analog of U0126	5, 10
ML-7	MLCK	5, 10
Y-27632	ROCK	5, 10

Genistein, ST638, FR180204 and ML-7 were purchased from Sigma-Aldrich, Burlington MA, USA. SCH772984 was bought from Cayman Chemical, Ann Arbor, MI, USA. PD98059 and Y-27632 were obtained from Tocris Bioscience, Avonmouth, Bristol UK. U0124 and U0126 were acquired from EMD Millipor Corporation, Billerica, MA, USA.

**Table 2:** Primary antibodies.

Antibody	Dilution	Purchased From	Catalog Number
ERK 1/ERK 2	1:3000	Cell Signaling Technology	9102S
p-ERK 1/ERK 2	1:3000	Cell Signaling Technology	9101S
MYPT 1	1:1000	Cell Signaling Technology	2634S
pThr-696-MYPT1	1:1000	Cell Signaling Technology	5163S

Cell Signaling Technology, Danvers, MA, USA.

## Data Availability

The original contributions presented in this study are included in the article. Further inquiries can be directed to the corresponding authors.

## Supplementary Materials

The following supporting information can be downloaded at https://www.mdpi.com/article/10.3390/biology14111557/s1: Figure S1. Airway smooth muscle stimulation with KCl 20 mM augments basal phosphorylation of ERK and MYPT1. Figure S2. Original Western blots used to construct densitometry analysis.

## Abbreviations

The following abbreviations are used in this manuscript:

ASM	Airway smooth muscle
CaM	Calmodulin
CaMK	Calcium calmodulin kinase
EGFR	Epidermal growth factor receptor
ERK	Epidermal growth factor receptor
GAP	GTPase-activating proteins
GEF	Nucleotide exchange factor
IEG	Immediate early genes
L-VDCC	L-type voltage dependent Ca^2+^ channels
MAPK	Mitogen-activated kinase
MEK	Mitogen-activated kinase kinase
MLCK	Myosin light chain kinase
MLCP	Myosin light chain phosphatase
MYPT1	Myosin phosphatase targeting subunit
ROCK	Rho kinase
RTK	Receptor tyrosine kinase
