# Supplementary material for: Characterization of the Signaling Pathways Activated by KCl-Induced RTK Stimulation in Guinea Pig Airways
Source: Biology (Basel). 2025 Nov 6;14(11):1557. doi: 10.3390/biology14111557 (PMC12650617; doi:10.3390/biology14111557)

Supplementary Figure S2. Original Western Blots used to construct desitometry analysis

FIGURE 1B ERK-P

n=1

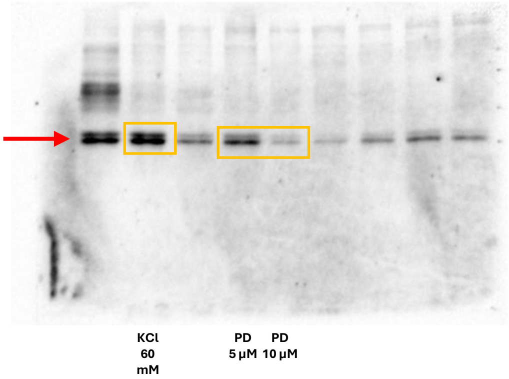

n=2

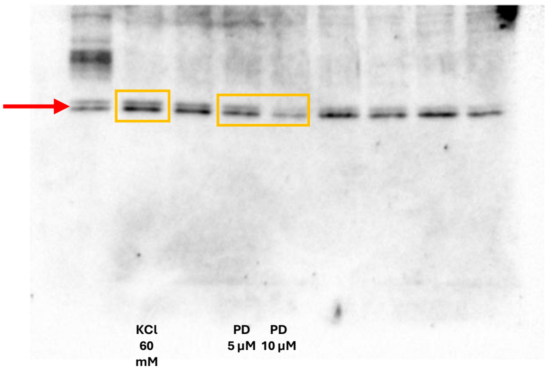

n=3

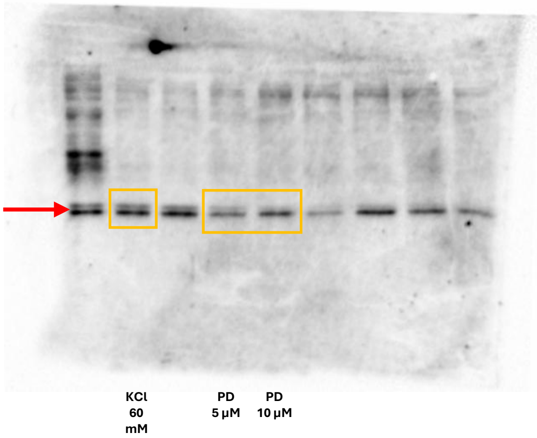

n=4

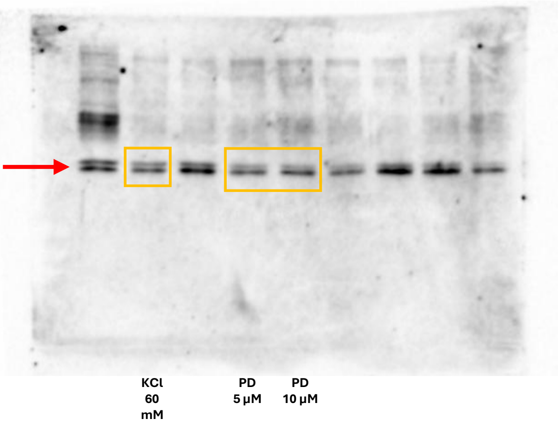

FIGURE 1B ERK-TOTAL

n=1

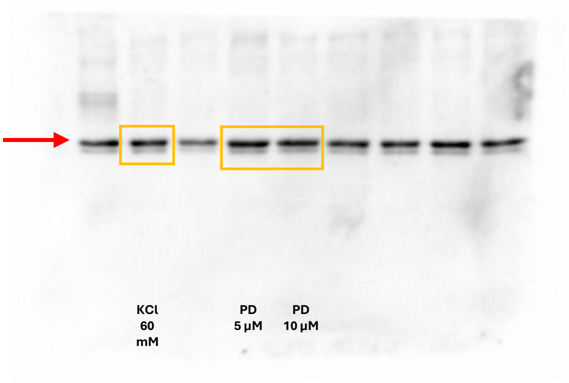

n=2

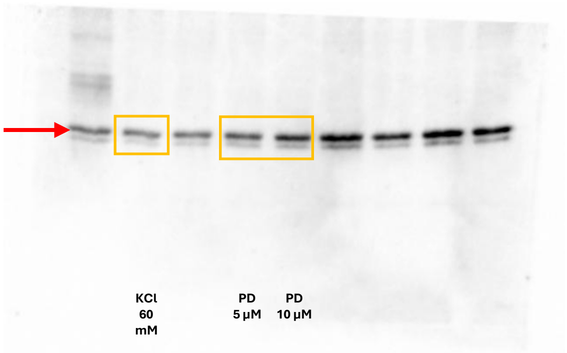

n=3

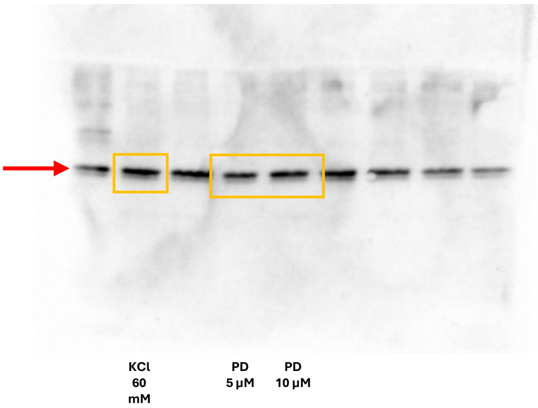

n=4

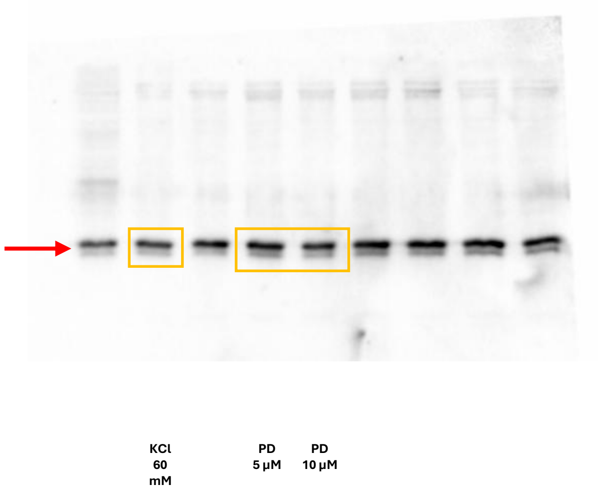

FIGURE 8A ERK-P

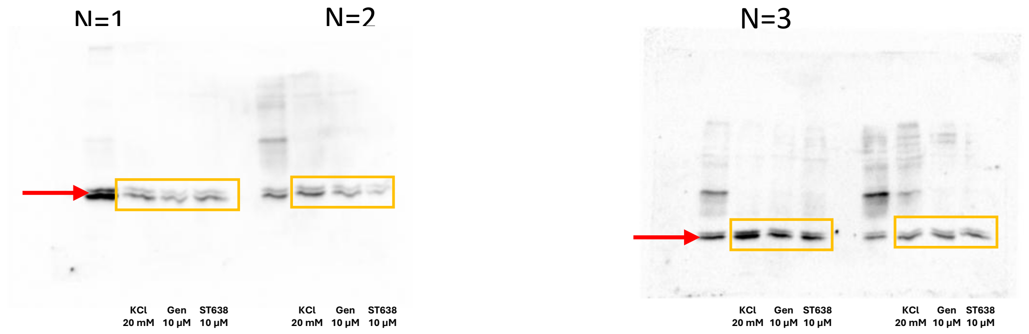

FIGURE 8A ERK-TOTAL

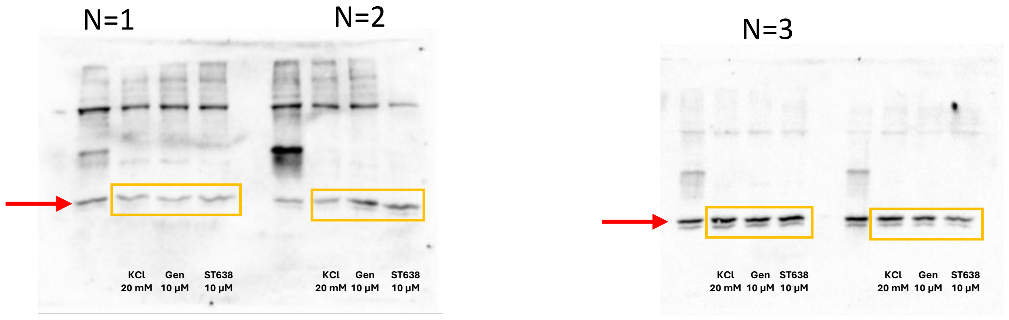

FIGURE 8B MYPT-P

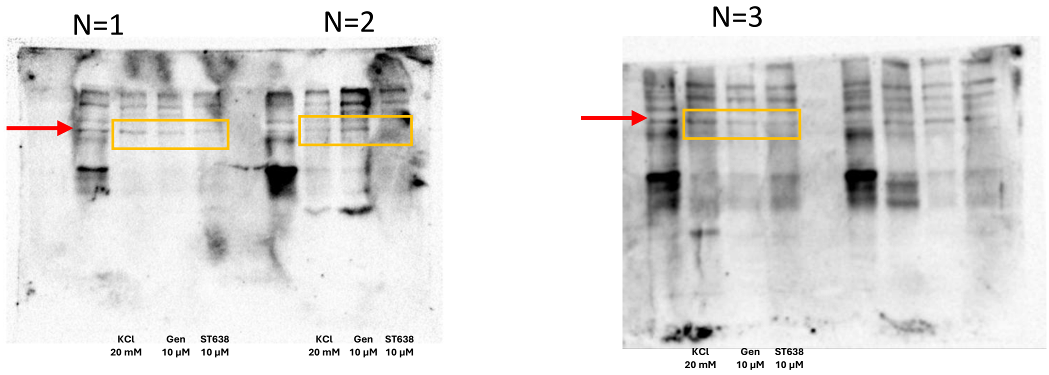

FIGURE 8B MYPT-TOTAL

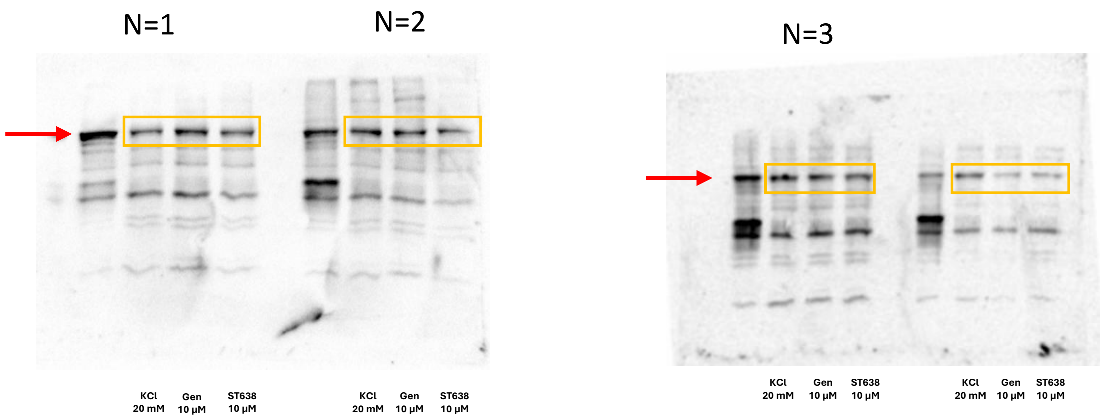

FIGURE 9A ERK-P

N=1 N=2 N=3

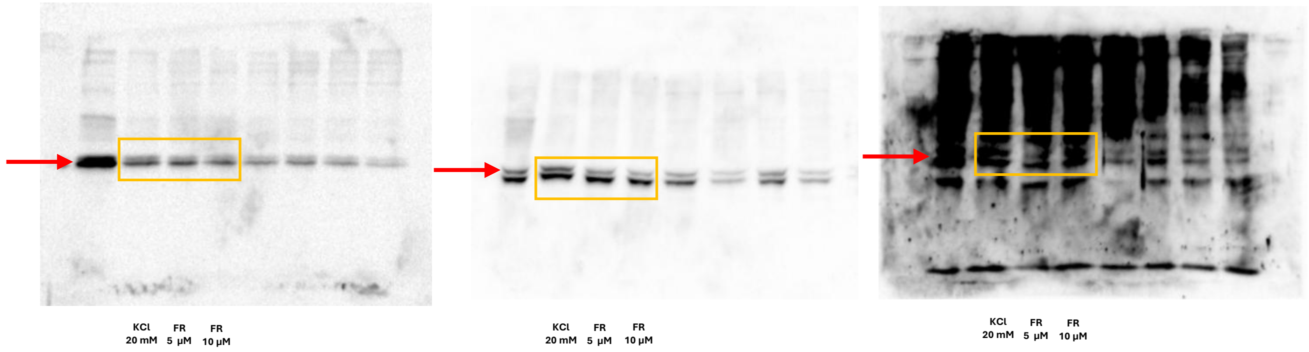

FIGURE 9A ERK-TOTAL

N=1 N=2 N=3

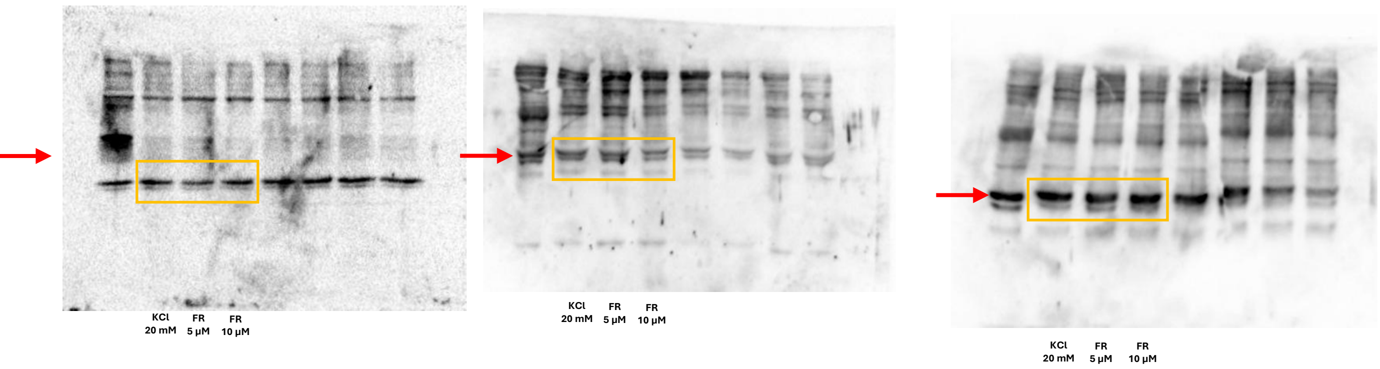

FIGURE 9B MYPT-P

N=1 N=2 N=3

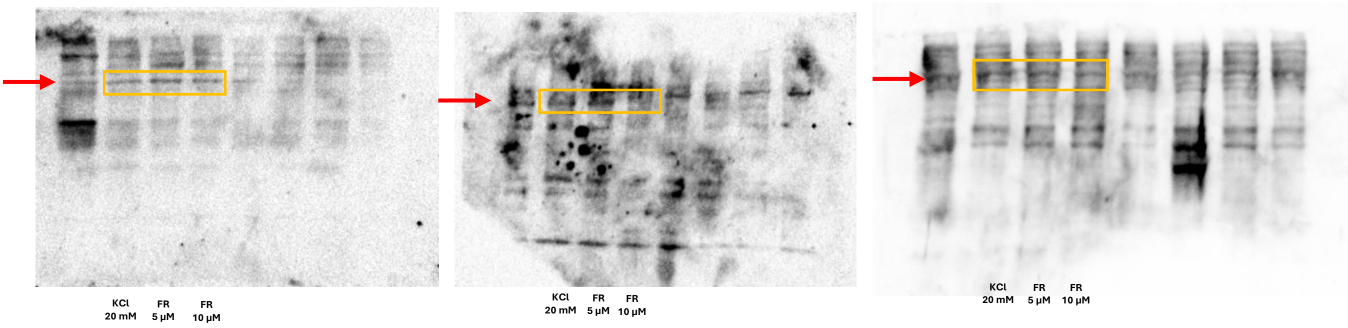

FIGURE 9B MYPT-TOTAL

N=1 N=2 N=3

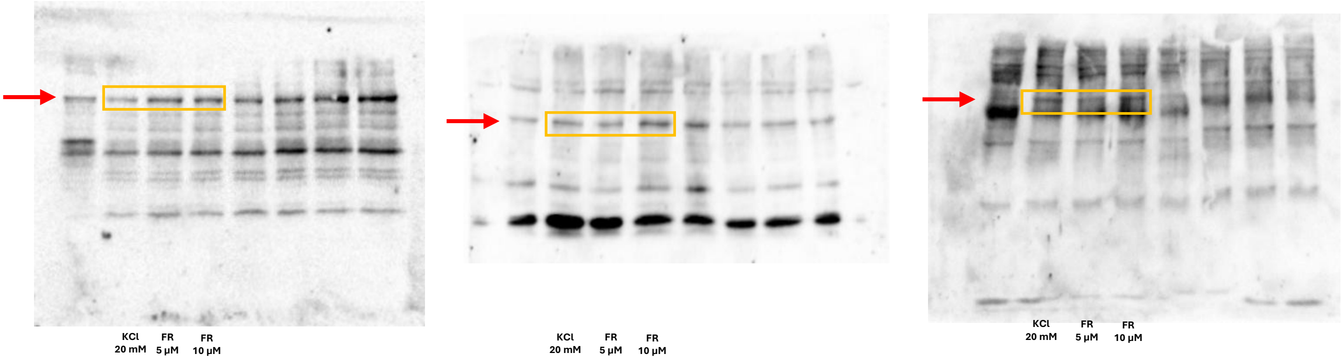

FIGURE FR180 32 µM   ERK-P

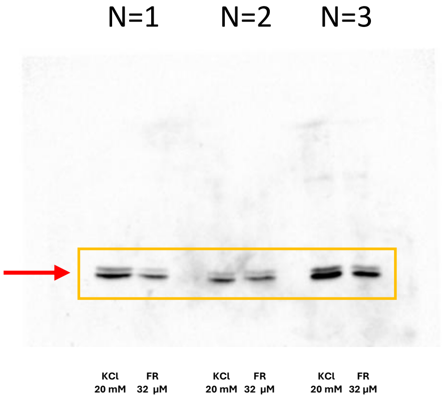

FIGURE FR180 ERK-TOTAL

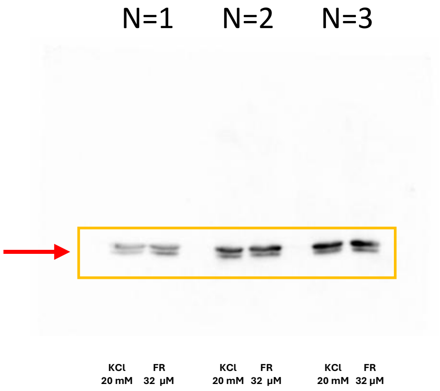

FIGURE SCH 32 µM   ERK-P

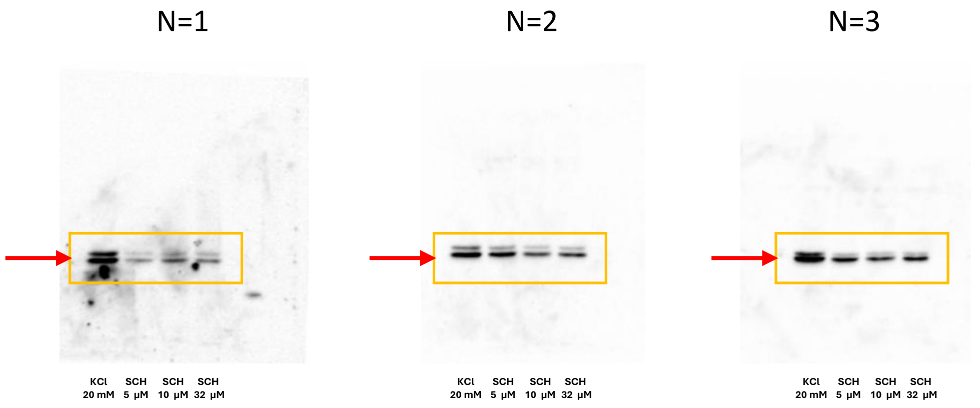

FIGURE SCH ERK-TOTAL

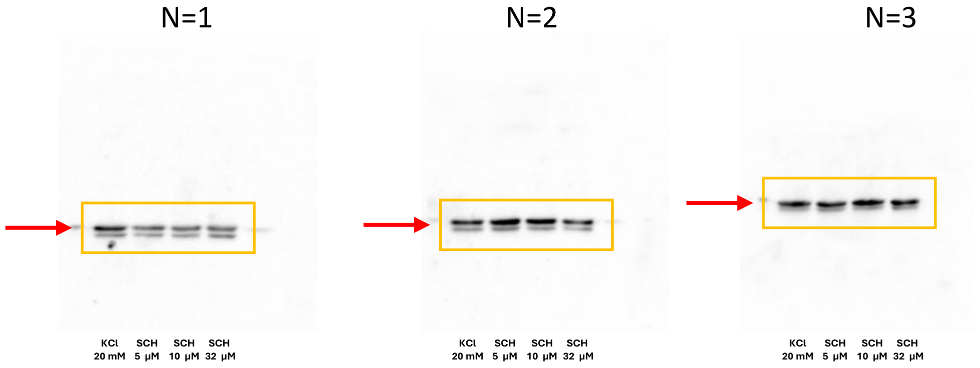

FIGURE 10A ERK-P

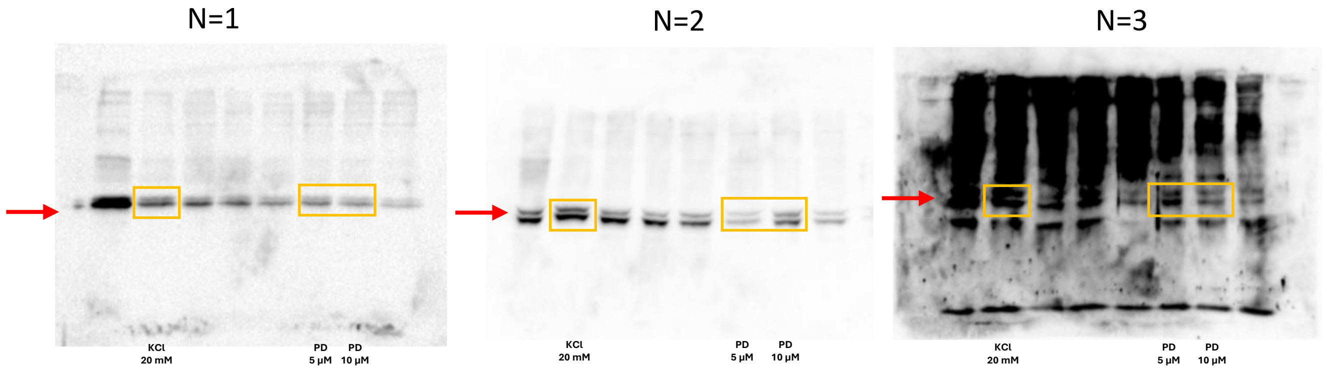

FIGURE 10A ERK-TOTAL

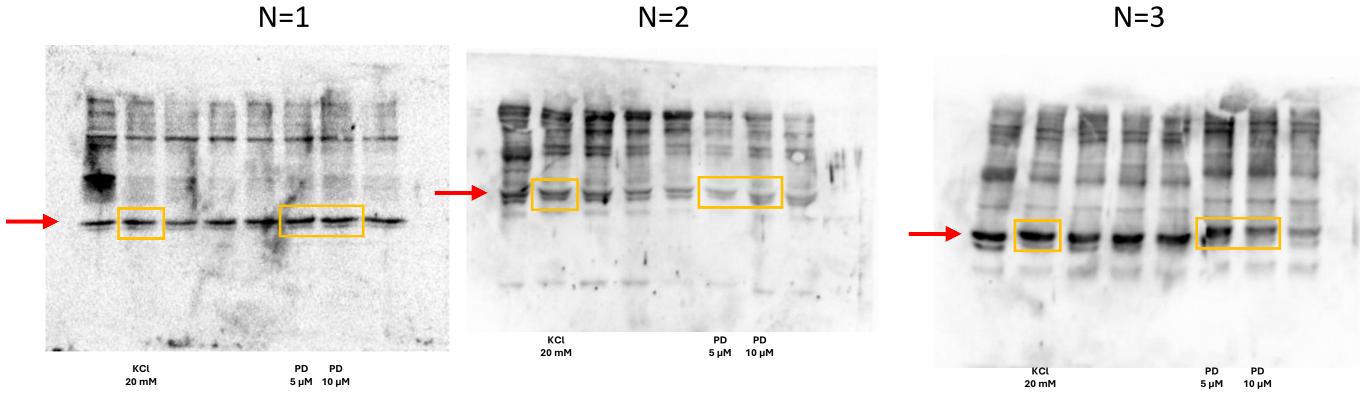

FIGURE 10B MYPT-P

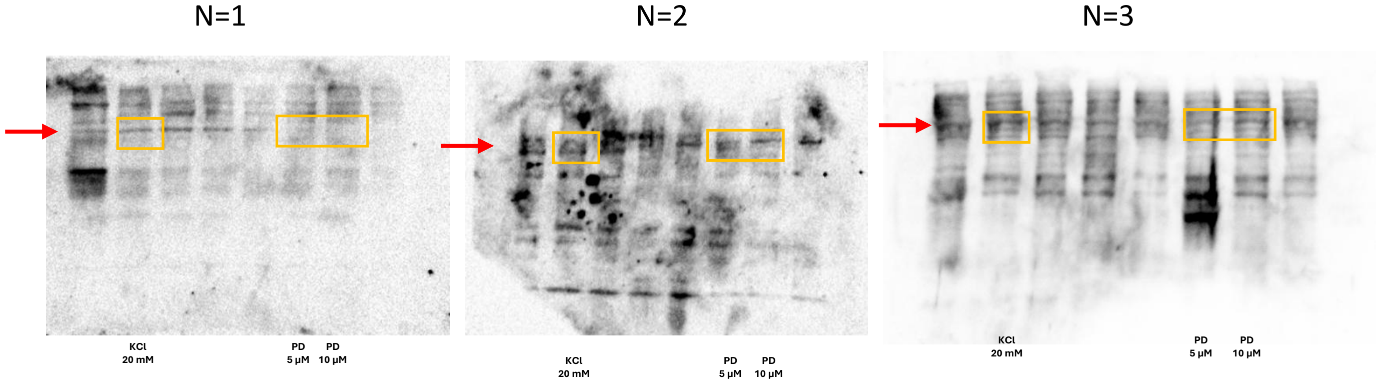

FIGURE 10B MYPT-TOTAL

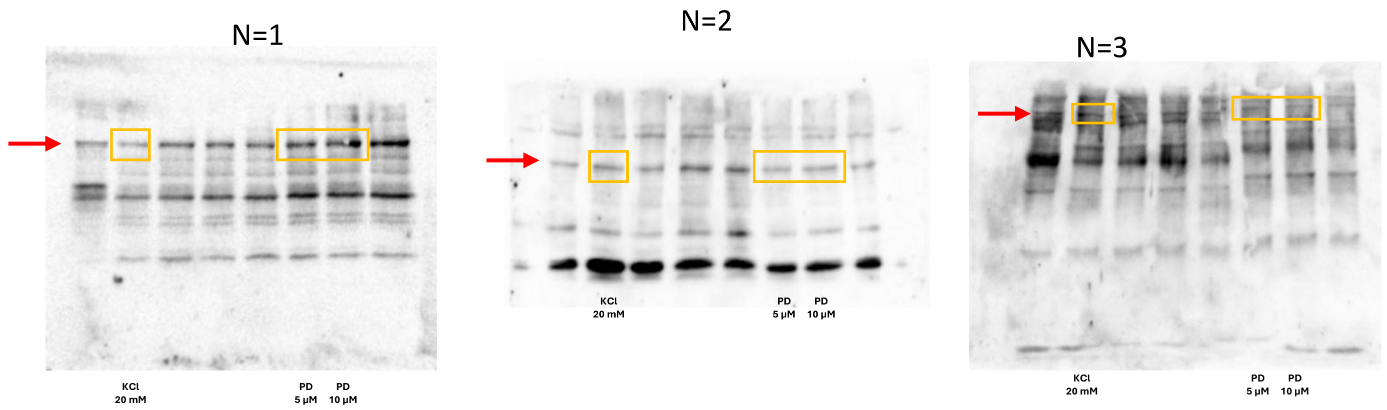

FIGURE 11A ERK-P

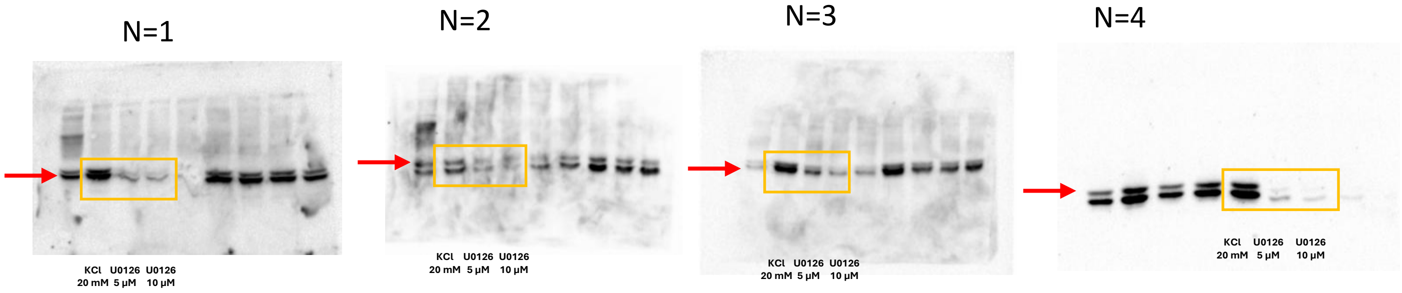

FIGURE 11A ERK-TOTAL

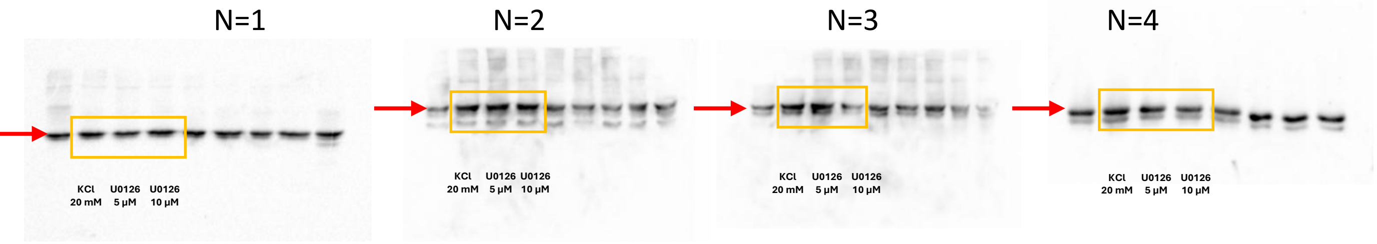

FIGURE 11B MYPT-P

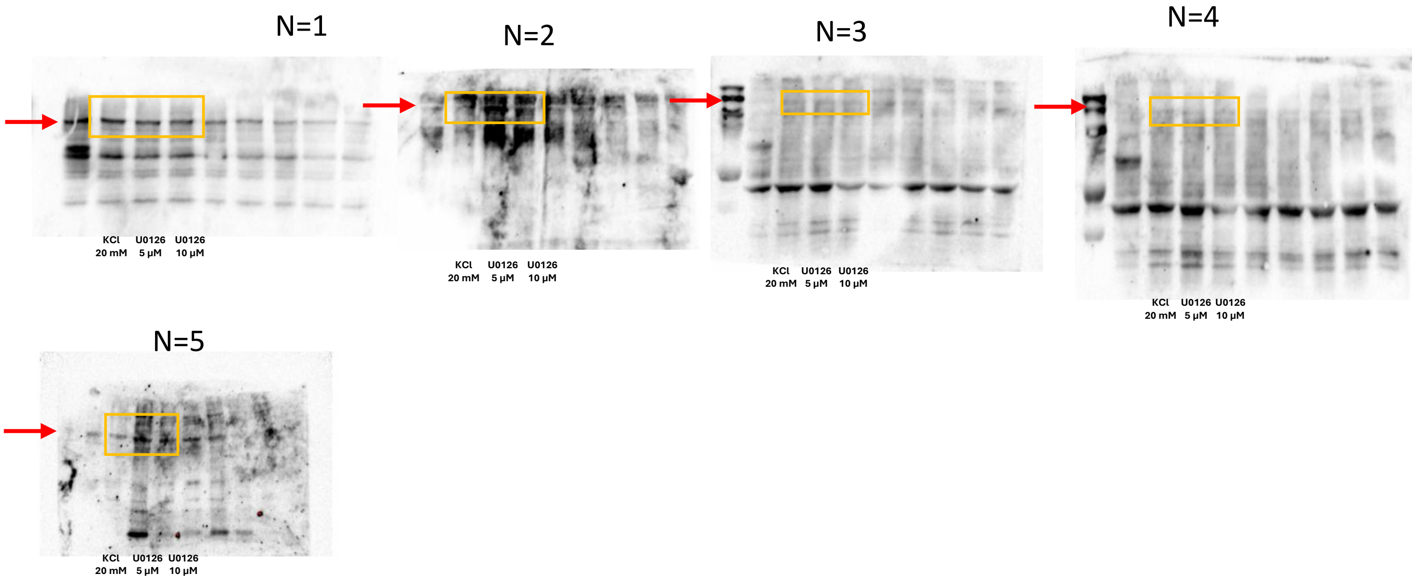

FIGURE 11B MYPT-TOTAL

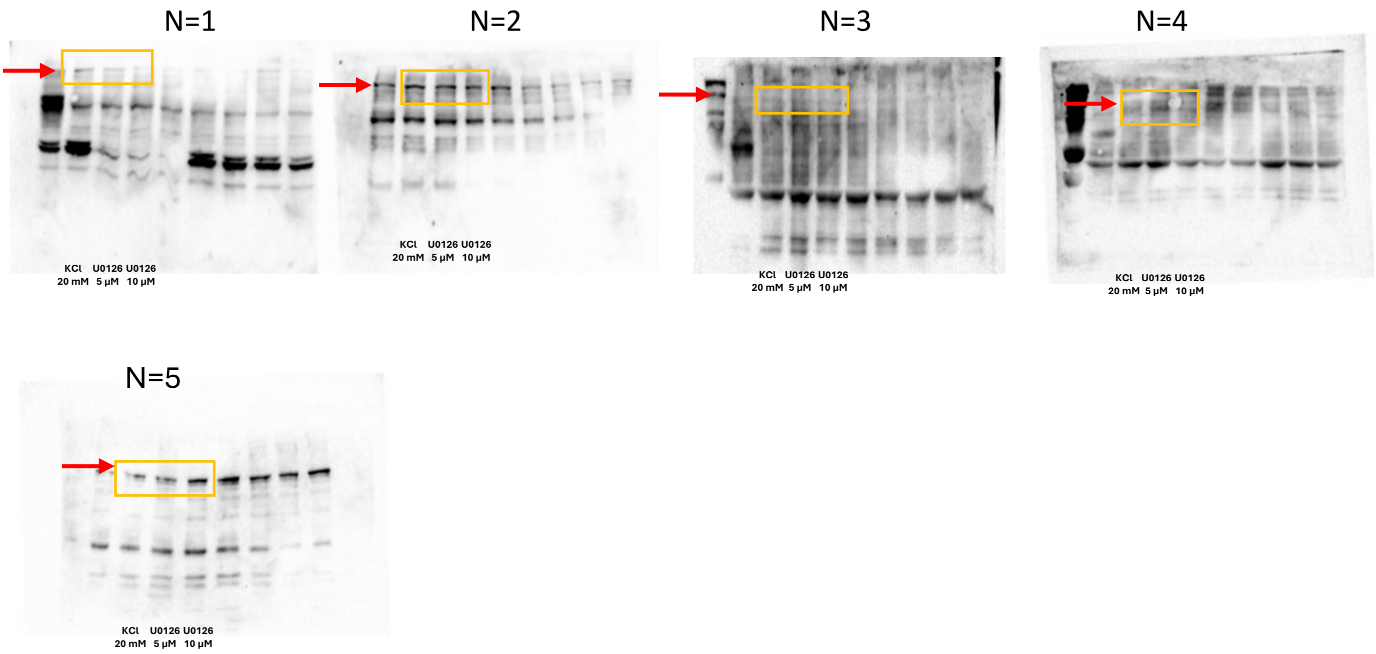

FIGURE 12A ERK-P

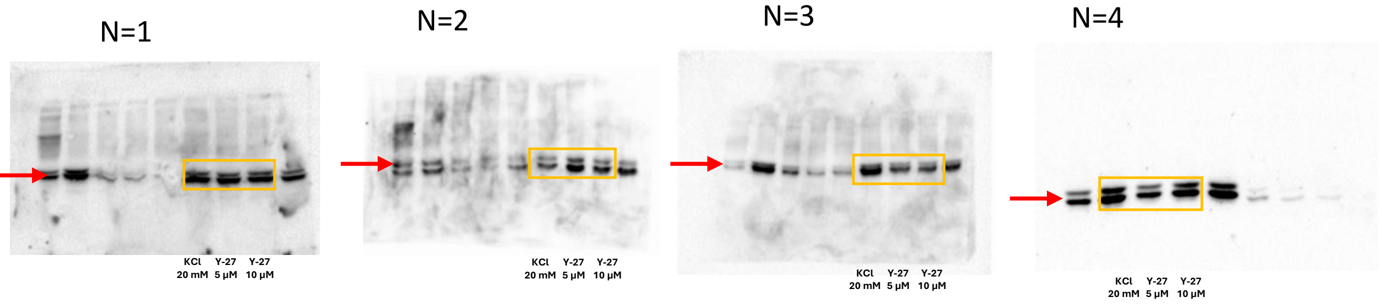

FIGURE 12A ERK-TOTAL

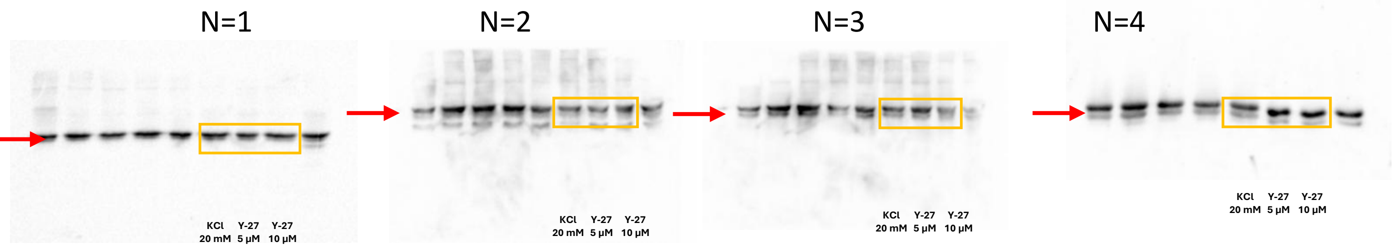

FIGURE 12B MYPT-P

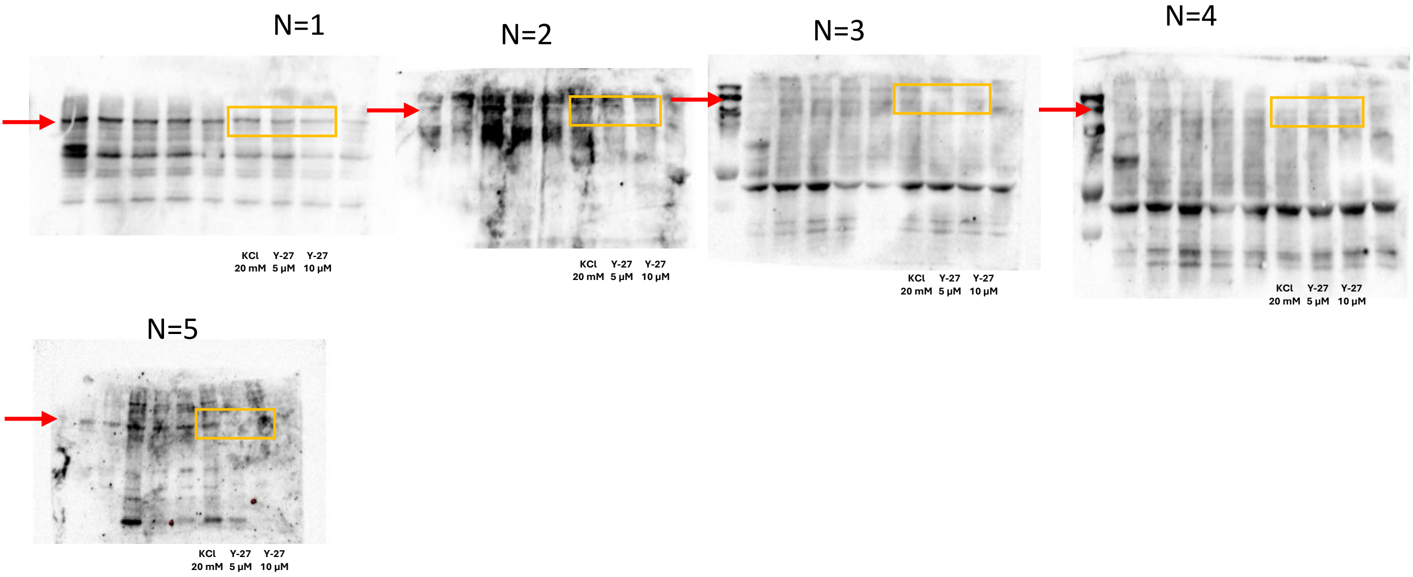

FIGURE 12B MYPT-TOTAL

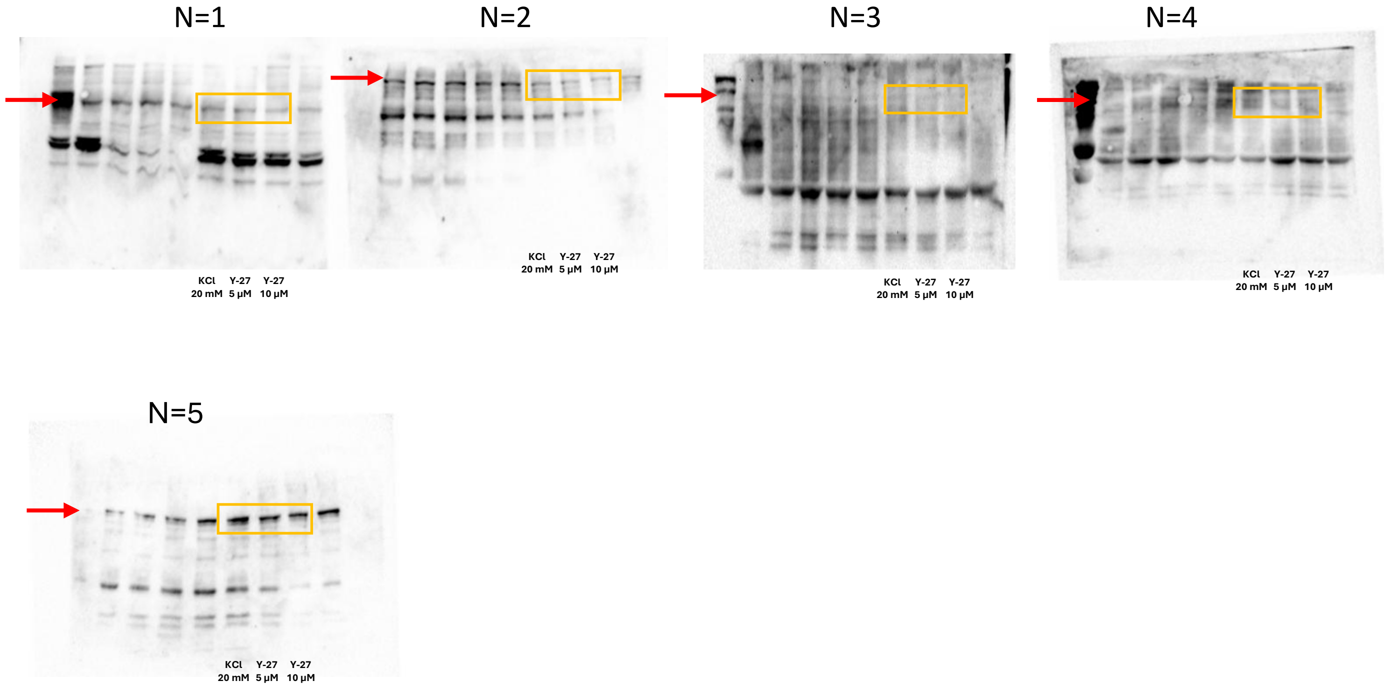

Supplement: Supplementary file 1 [file biology-14-01557-s001.zip › Suplementary Figure S2.pdf]
