# Supplementary material for: Characterization of the Signaling Pathways Activated by KCl-Induced RTK Stimulation in Guinea Pig Airways
Source: Biology (Basel). 2025 Nov 6;14(11):1557. doi: 10.3390/biology14111557 (PMC12650617; doi:10.3390/biology14111557)

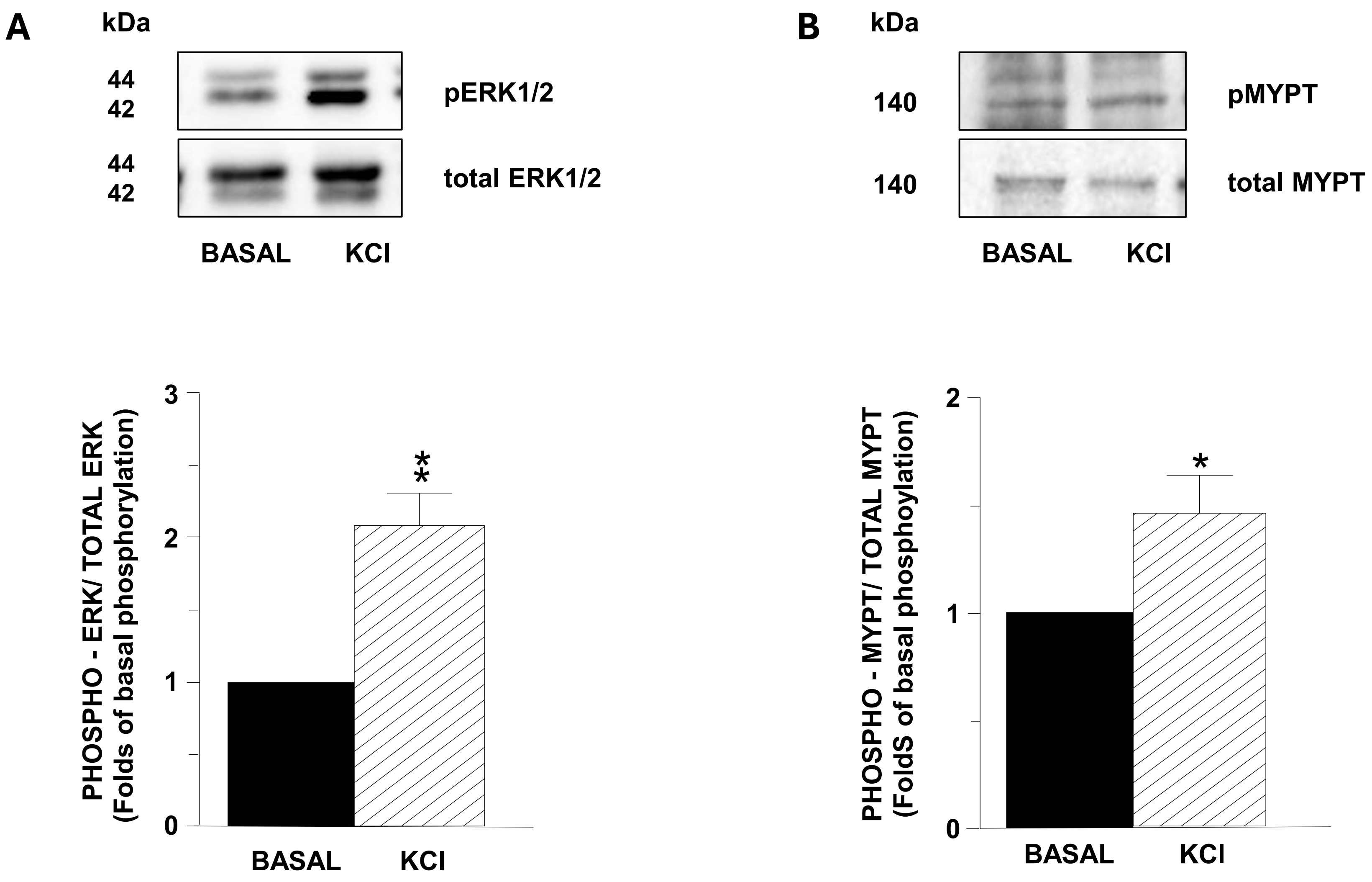

Supplementary Figure S1. Airway smooth muscle stimulation with KCl 20 mM augments basal phosphorylation of **A)**ERK (n=3, **$p<0.01$) and **B)**MYPT1 (n=4, $p<0.05$).

Uncropped blots

pERK1/2

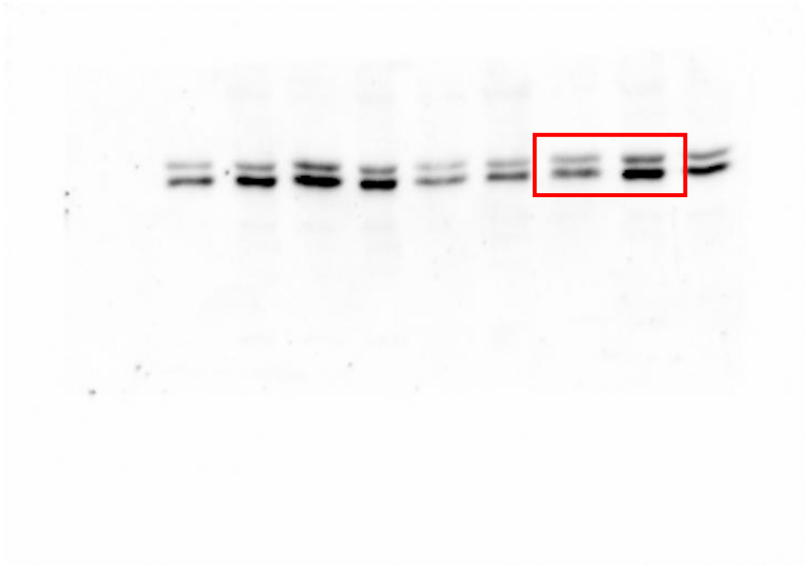

total ERK1/2

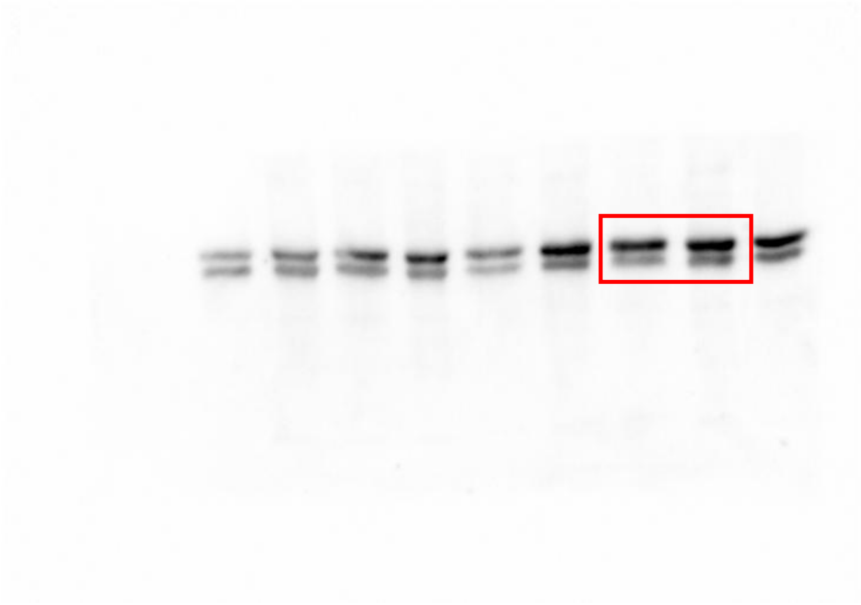

pMYPT1

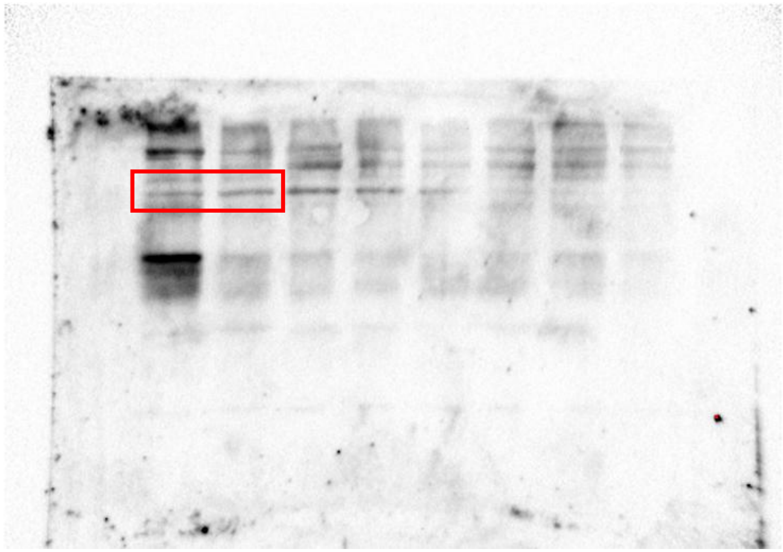

total MYPT1

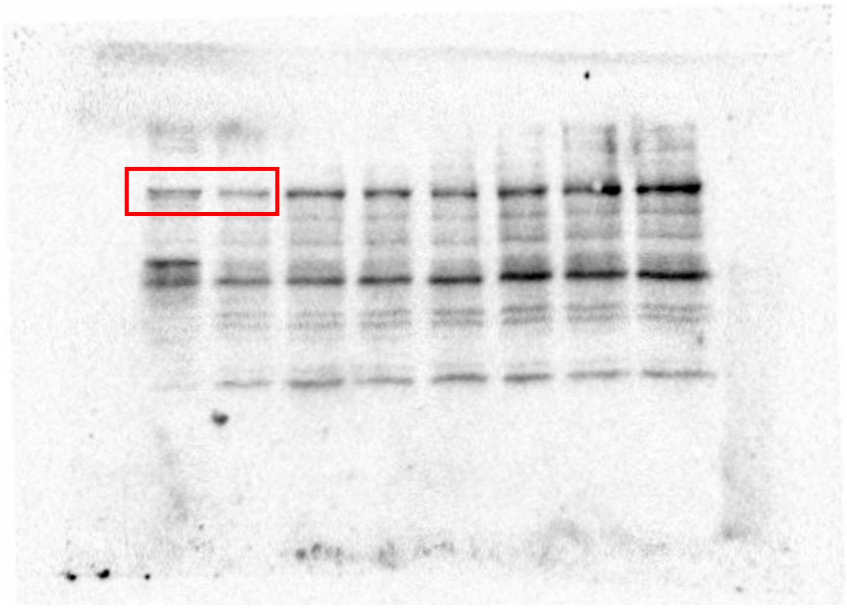

Supplement: Supplementary file 1 [file biology-14-01557-s001.zip › Supplementary Figure S1.pdf]
